# Luminal breast cancer identity is determined by loss of glucocorticoid receptor activity

**DOI:** 10.15252/emmm.202317737

**Published:** 2023-10-30

**Authors:** Stefan Prekovic, Theofilos Chalkiadakis, Merel Roest, Daniel Roden, Catrin Lutz, Karianne Schuurman, Mark Opdam, Liesbeth Hoekman, Nina Abbott, Tanja Tesselaar, Maliha Wajahat, Amy R Dwyer, Isabel Mayayo‐Peralta, Gabriela Gomez, Maarten Altelaar, Roderick Beijersbergen, Balázs Győrffy, Leonie Young, Sabine Linn, Jos Jonkers, Wayne Tilley, Theresa Hickey, Damir Vareslija, Alexander Swarbrick, Wilbert Zwart

**Affiliations:** ^1^ Division of Oncogenomics, Oncode Institute The Netherlands Cancer Institute Amsterdam The Netherlands; ^2^ Center for Molecular Medicine UMC Utrecht Utrecht The Netherlands; ^3^ Cancer Ecosystems Program Garvan Institute of Medical Research Darlinghurst NSW Australia; ^4^ School of Clinical Medicine, Faculty of Medicine and Health UNSW Sydney Sydney NSW Australia; ^5^ Division of Molecular Pathology, Oncode Institute The Netherlands Cancer Institute Amsterdam The Netherlands; ^6^ Mass Spectrometry/Proteomics Facility The Netherlands Cancer Institute Amsterdam The Netherlands; ^7^ Dame Roma Mitchell Cancer Research Laboratories, Adelaide Medical School University of Adelaide Adelaide SA Australia; ^8^ School of Pharmacy and Biomolecular Sciences The Royal College of Surgeons University of Medicine and Health Sciences Dublin Ireland; ^9^ Biomolecular Mass Spectrometry and Proteomics, Bijvoet Center for Biomolecular Research and Utrecht Institute for Pharmaceutical Sciences Utrecht University Utrecht The Netherlands; ^10^ Division of Molecular Carcinogenesis and Robotics and Screening Centre Netherlands Cancer Institute Amsterdam The Netherlands; ^11^ TTK Cancer Biomarker Research Group Institute of Enzymology Budapest Hungary; ^12^ Department of Bioinformatics and 2nd Department of Pediatrics Semmelweis University Budapest Hungary; ^13^ Endocrine Oncology Research Group, Department of Surgery The Royal College of Surgeons University of Medicine and Health Sciences Dublin Ireland; ^14^ Beaumont RCSI Cancer Centre Beaumont Hospital Dublin Ireland; ^15^ Freemasons Centre for Male Health and Wellbeing University of Adelaide Adelaide SA Australia; ^16^ Laboratory of Chemical Biology and Institute for Complex Molecular Systems, Department of Biomedical Engineering Eindhoven University of Technology Eindhoven The Netherlands

**Keywords:** breast cancer, glucocorticoids, luminal breast cancer subtypes, nuclear receptors, ZBTB16, Biomarkers, Cancer, Chromatin, Transcription & Genomics

## Abstract

Glucocorticoid receptor (GR) is a transcription factor that plays a crucial role in cancer biology. In this study, we utilized an *in silico*‐designed GR activity signature to demonstrate that GR relates to the proliferative capacity of numerous primary cancer types. In breast cancer, the GR activity status determines luminal subtype identity and has implications for patient outcomes. We reveal that GR engages with estrogen receptor (ER), leading to redistribution of ER on the chromatin. Notably, GR activation leads to upregulation of the *ZBTB16* gene, encoding for a transcriptional repressor, which controls growth in ER‐positive breast cancer and associates with prognosis in luminal A patients. In relation to *ZBTB16*'s repressive nature, GR activation leads to epigenetic remodeling and loss of histone acetylation at sites proximal to cancer‐driving genes. Based on these findings, epigenetic inhibitors reduce viability of ER‐positive breast cancer cells that display absence of GR activity. Our findings provide insights into how GR controls ER‐positive breast cancer growth and may have implications for patients' prognostication and provide novel therapeutic candidates for breast cancer treatment.

## Graphical abstract

Activity of the glucocorticoid receptor across various cancer types is inversely related to proliferative signatures. In breast cancer, heightened glucocorticoid receptor (GR) activity correlates with improved patient outcomes and is linked to the regulation of the tumor suppressor ZBTB16.

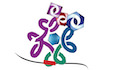

**The paper explained**

**Problem**

The glucocorticoid receptor (GR) is known for its significant role in cancer biology. However, its impact on proliferative capacity across cancer types, and among breast cancer subtypes, remains a key area to explore.

**Results**

Utilizing an *in silico*‐designed GR activity signature, this study explores the relationship of GR with proliferative capacity in 33 cancer types. Focusing on breast cancer, GR activity status defines luminal subtype identity and is associated with favorable patient outcomes. GR is specifically active in Luminal A tumors, where it interacts with the estrogen receptor (ER); it redistributes ER on the chromatin and upregulates the transcriptional repressor gene ZBTB16. ZBTB16 controls growth in ER‐positive breast cancer and is associated with a favorable prognosis in luminal A patients. Moreover, GR activation leads to significant epigenetic remodeling and decreased histone acetylation of cancer‐driving gene loci. Using epigenetic drugs, we provide pre‐clinical proof of concept targeting both luminal A and B breast cancer cells.

**Impact**

This research provides a comprehensive assessment of GR action in cancer biology and its impact on tumor cell proliferation capacity, particularly in ER‐positive breast cancer. It offers novel avenues for improved patient prognostication and introduces potential new therapeutic candidates for effective breast cancer treatment.

## Introduction

The glucocorticoid receptor (GR) is a transcription factor that regulates gene expression in response to glucocorticoids (Desmet & De Bosscher, 2017). Its role in solid cancers is not fully understood, and the consequences of its signaling within cancer cells remain unclear. While systemic glucocorticoid treatment leads to immunosuppression, the implications of GR activity in cancer cells are multifaceted and context dependent. GR can act as both a tumor suppressor and an oncogene, depending on the cancer type or disease stage (Arora *et al*, 2013; Terwilliger & Abdul‐Hay, 2017; Obradović *et al*, 2019; Prekovic *et al*, 2021; Mayayo‐Peralta *et al*, 2021b). This poses a potential constraint in terms of cancer treatment and progression. Therefore, understanding the biology of GR in solid cancers is critical for identifying potential biomarkers and therapeutic targets.

Glucocorticoids (GCs), such as dexamethasone, are an essential component in breast cancer management due to their therapeutic efficacy in mitigating chemotherapy‐induced side effects and addressing advanced‐stage symptoms (Vaidya *et al*, 2010). While the expression of GR is decreased during breast cancer development (Perou *et al*, 2000; Sørlie *et al*, 2001; Lien *et al*, 2006; Conde *et al*, 2008; Buxant *et al*, 2010), studies have shown that GR activation by GCs can inhibit cell proliferation in estrogen receptor (ER)‐positive breast cancer (Tonsing‐Carter *et al*, 2019). This negative regulation is believed to be mediated through direct inhibition of ER enhancer function (Yang *et al*, 2017). Conversely, the role of GR in ER‐negative breast cancer is more complex, with studies suggesting that it may support cancer growth and metastasis, aggravating clinical aggressiveness (Pan *et al*, 2011; West *et al*, 2018). Activation of GR in ER‐negative disease supports an epithelial–mesenchymal transition (EMT) gene program and is associated with shorter relapse‐free survival (Pan *et al*, 2011). Recent studies have linked GR activation to EMT processes in ER‐negative breast cancer and demonstrated that it can increase colonization and reduce survival of animal models (Obradović *et al*, 2019). These findings suggest that GR antagonism may increase chemotherapy efficacy in ER‐negative breast cancers and inhibit metastatic spread, although this has yet to be tested clinically (Prekovic & Zwart, 2023).

In this study, we aimed to elucidate the intricate and diverse role of GR as a transcription factor in cancer biology, specifically focusing on breast cancer. Acknowledging that GR's role in breast cancer is contingent on factors such as cancer subtype, disease stage, and treatment approach, we aimed to better understand GR biology in this context. Employing an *in silico*‐designed GR activity signature, we illustrated GR's regulatory impact on the proliferative potential of a wide range of different primary cancer types. Our investigation explores the relationship among GR activity status, ER signaling, and luminal subtype identity, with implications for patient outcomes in breast cancer. Importantly, we identified *ZBTB16* as a glucocorticoid‐driven suppressor of ER‐positive breast cancer growth and demonstrated that clinically available epigenetic inhibitors effectively impede the growth of ER‐positive breast cancer cells without an active GR. Our findings offer valuable insights into GR's action in ER‐positive breast cancer, with potential future implications for patient prognostication and therapeutic strategy optimization.

## Results

### GR activity is related to proliferative capacity of numerous primary cancer types

**Figure 1:**
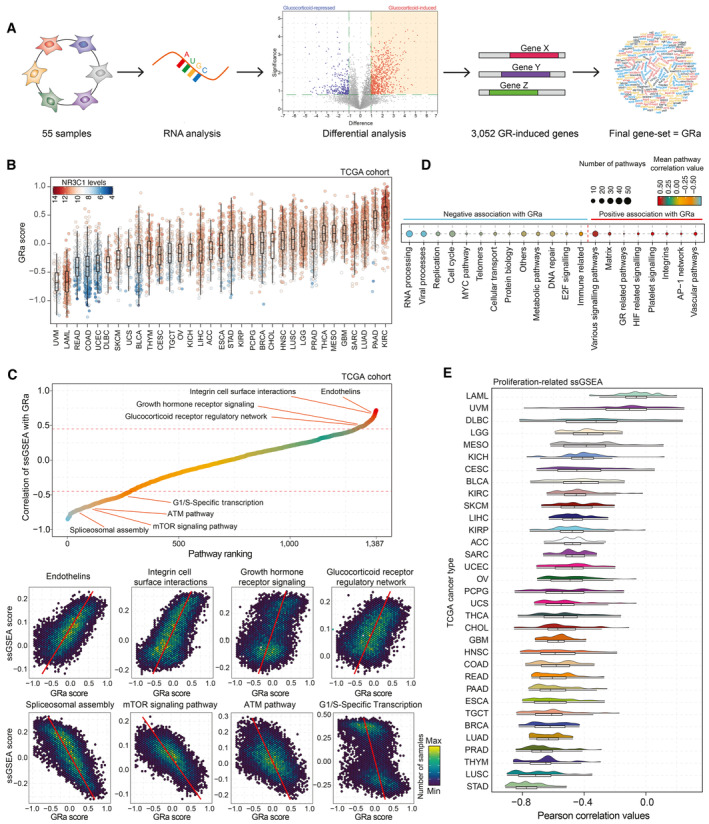
Exploration of GR functions in different primary cancers Schematic representation of GR activity signature development.GR activity calculations for sample of the TCGA cohort (*n* = 9,874) split by cancer type. Color of each data point represents NR3C1 mRNA levels (red, white, and blue represent high, intermediate, and low levels, respectively). The box begins in the first quartile (25%) and ends in the third (75%), while the line represents the median value. The lines represent segments to furthest data without accounting for outliers.(top) Correlation analysis of single‐sample gene‐set enrichment analysis (ssGSEA) scores calculated per TCGA sample (*n* = 9,829), and GR activity, with several top pathways highlighted. (bottom) Pearson correlation plots depicting pathway scores and GR activity for pathways highlighted in the panel above. Each hex represents a group of patients with similar values, and the color of the hex represents how many samples fall into that particular space.Significantly correlating single‐sample GSEA families (number of pathways grouped is reflected by the point size) with the average correlation with GR activity signature reported as a color gradient. Only pathway families with strong Pearson correlation are displayed (*R* < −0.45 or > 0.45).Distribution of Pearson correlation values for all the proliferation‐related ssGSEA signatures and GR activity for each TCGA tumor type independently. Under the distribution graph, a boxplot is depicted. The box itself spans from the first quartile (25%) to the third quartile (75%), representing the interquartile range where the central 50% of data values fall. Inside the box, a line denotes the median value. The whiskers of the boxplot extend from the ends of the box to the minimum and maximum values.

**Figure EV1:**
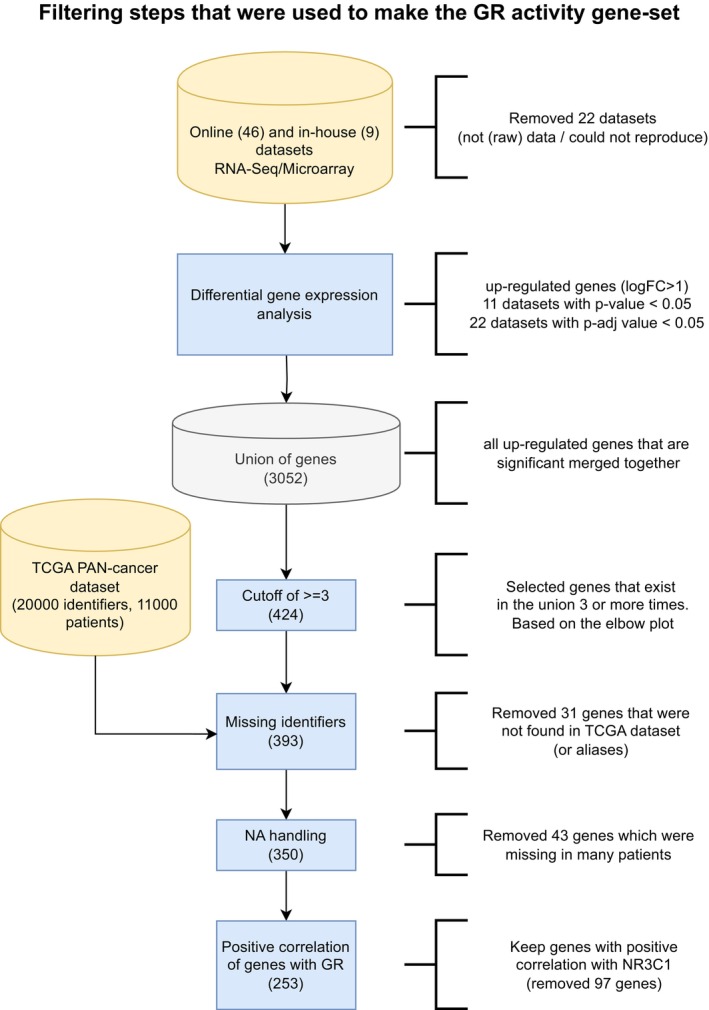
The scheme representing the process and filtering steps taken to make the GR activity signature

**Figure EV2:**
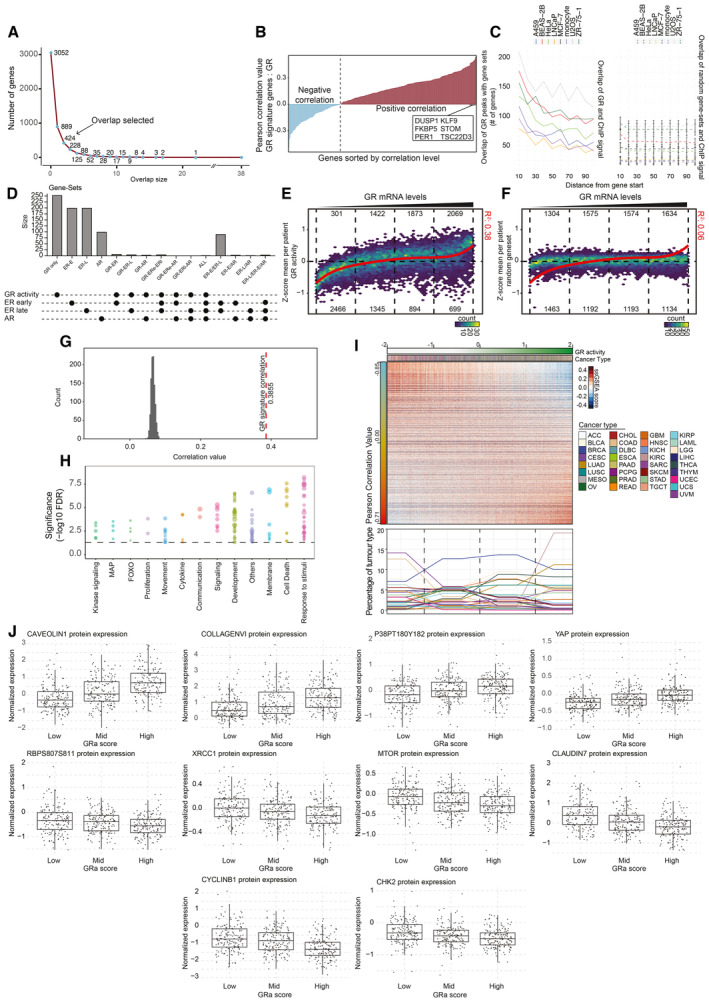
Optimization of the GR activity signature Elbow plot depicting overlap between models. This analysis aided refinement of the signature to a consensus list of number of genes shared among at least three independent models, yielding a total list of 424 genes.Expression correlation analyses of NR3C1 mRNA levels with expression levels of genes represented in the signature in cancer samples. Only positively correlating genes were included for further analyses, yielding a final set of 253 genes.Enrichment of GR binding in vicinity of GRa (right, full line) or random (left, dashed line; *n* = 1,000 iterations; error bars represent mean ± SD) genes in ChIP‐sequencing experiments from various cell lines (full lines, A549, BEAS‐2B, HeLa, LNCaP, MCF‐7, THP1, U2OS, and ZR‐75‐1).Overlap of GR activity signature, with ER (gene‐set reference: M5906 and M5907) and AR (gene‐set reference: M5908) gene signatures.Correlation of NR3C1 mRNA levels with GR activity across samples represented in the TCGA dataset. Color indicates the number of samples in each bin.Correlation of GR mRNA levels with random gene set of equivalent size to GR activity signature among samples represented in the TCGA dataset. Color indicates the number of samples in each bin.Histogram depicting correlation of random gene set of equivalent size to GR activity signature with NR3C1 mRNA levels in TCGA cancers; black line depicts the correlation of GR activity with NR3C1 mRNA levels in the same cohort.Overrepresentation analysis of various gene sets within the GR activity signature. Each point represents a different pathway.Pearson correlation analysis of single‐sample gene‐set enrichment analysis (ssGSEA) scores for each of the TCGA samples (*n* = 9,829) and GR activity. Representation of the different tumor types is depicted below.Normalized (phospho)protein expression in human breast cancer samples of the TCGA (*n* = 747) grouped based on GR activity. The central mark indicates the median, and the bottom and top edges of the box indicate the 25^th^ and 75^th^ percentiles, respectively. The maximum whisker lengths are specified as 1.5 times the interquartile range.

To study GR activity, and the consequences thereof, across different primary cancer types, we made use of 55 *in vitro* experimental models that were exposed to glucocorticoids (e.g., dexamethasone (Dexa) and hydrocortisone) or vehicle (Veh), and subsequently processed for transcriptome profiling (Fig 1A; a more detailed overview can be found in Fig EV1; Details on models and public accession codes can be found in Table EV1). Following independent, per‐model, differential analysis, we created a union of genes upregulated by glucocorticoids across all the models (union size = 3,052). To develop a smaller, more reliable signature, we exclusively selected those genes that were identified in a minimal overlap of three models (Fig EV2A) and with positive Pearson correlation value with *NR3C1* (encodes for GR protein) mRNA levels in The Cancer Genome Atlas cohort (TCGA; https://www.cancer.gov/tcga; Fig EV2B, *R* > 0). This produced a gene set that we defined as GR activity (GRa) signature, containing 253 genes (Table EV2), which featured an enrichment of GR‐chromatin occupancy in their vicinity (100 kb analyzed; 10 kb increments were inspected), in comparison to random gene sets of the same size across various *in vitro* models (Fig EV2C). Importantly, GRa signature showed minimal overlap with previously established gene sets related to other steroid–hormone receptors with comparable DNA‐binding profiles—estrogen receptor (ER) (gene‐set reference: M5906 and M5907) and androgen receptor (gene‐set reference: M5908) (Fig EV2D). Across the TCGA cohort, we show that GRa only weakly correlates with *NR3C1* mRNA levels itself (Fig EV2E; *R* = 0.38), that is, at low ends of GR mRNA expression, the major fraction of all cancers have low GR activity, while for intermediate and high GR mRNA levels, a large variation exists between samples (Fig EV2E). The observed, although weak, correlation exceeds noise level, as seen by random gene‐set analysis (Fig EV2F and G, *n* = 1,000 iterations). Ultimately, we also explored potential contributions of other gene sets in our GRa signature showing that no particular signature by itself explains the genes found in the GRa gene set (Fig EV2H).

We next sought to explore the activity of GR across each of the 33 tumor types of the TCGA cohort, using bulk RNA‐sequencing data (Fig 1B). This analysis was followed up by single‐sample gene‐set enrichment analysis (ssGSEA; PARADIGM pathways (*n* = 1,387)) across all the cancer samples (*n* = 9,829) and subsequent correlation analysis with GRa (Fig EV2I). We identified individual pathways that associate with activity of GR, either negatively (e.g., ATM pathway and mTOR signaling; Fig 1C) or positively (e.g., growth hormone receptor signaling and endothelins; Fig 1C). The individual pathways were next grouped in families based on biological processes they correspond to, and the average correlation value was calculated (Fig 1D). Among the pathway families that positively correlated with GRa, we identified GR‐related pathways, as well as AP‐1 network (well‐known to cross‐talk with GR; Herrlich, 2001), increasing the confidence in our analysis (Fig 1D). In terms of pathway families that are negatively associated with GRa, we identified immunity‐associated (as GR is related to systemic immunosuppression; Desmet & De Bosscher, 2017) and metabolism‐related (as relates GR to metabolic regulation; Shimizu *et al*, 2011; Loft *et al*, 2022) gene sets, as well as genes linked to cell proliferation (Fig 1D). Next, we performed correlation analyses for proliferation‐related pathways relative to GRa, for each individual cancer type of the TCGA. Importantly, for the majority of primary cancers, GRa was negatively associated with proliferation‐related gene signatures (Fig 1E). Among the cancers with most‐negative correlation, we observed associations with lung adenocarcinoma (in line with our previous work; Prekovic *et al*, 2021) and breast cancer (Fig 1E; LUAD = lung adenocarcinoma, and BRCA = breast cancer). To further substantiate above‐mentioned findings, focusing on breast cancer, we inspected protein expression/phosphorylation status of proteins involved in cell cycle (cyclin B1), metabolism (MTOR and RPBS807S811), DNA damage response (XRCC1 and CHK2), matrix biology (collagen VI), or previously reported direct GR targets (caveolin 1), confirming the mRNA‐based observations (Fig EV2J).

### Breast cancer luminal identity is determined by GR activity status

**Figure 2:**
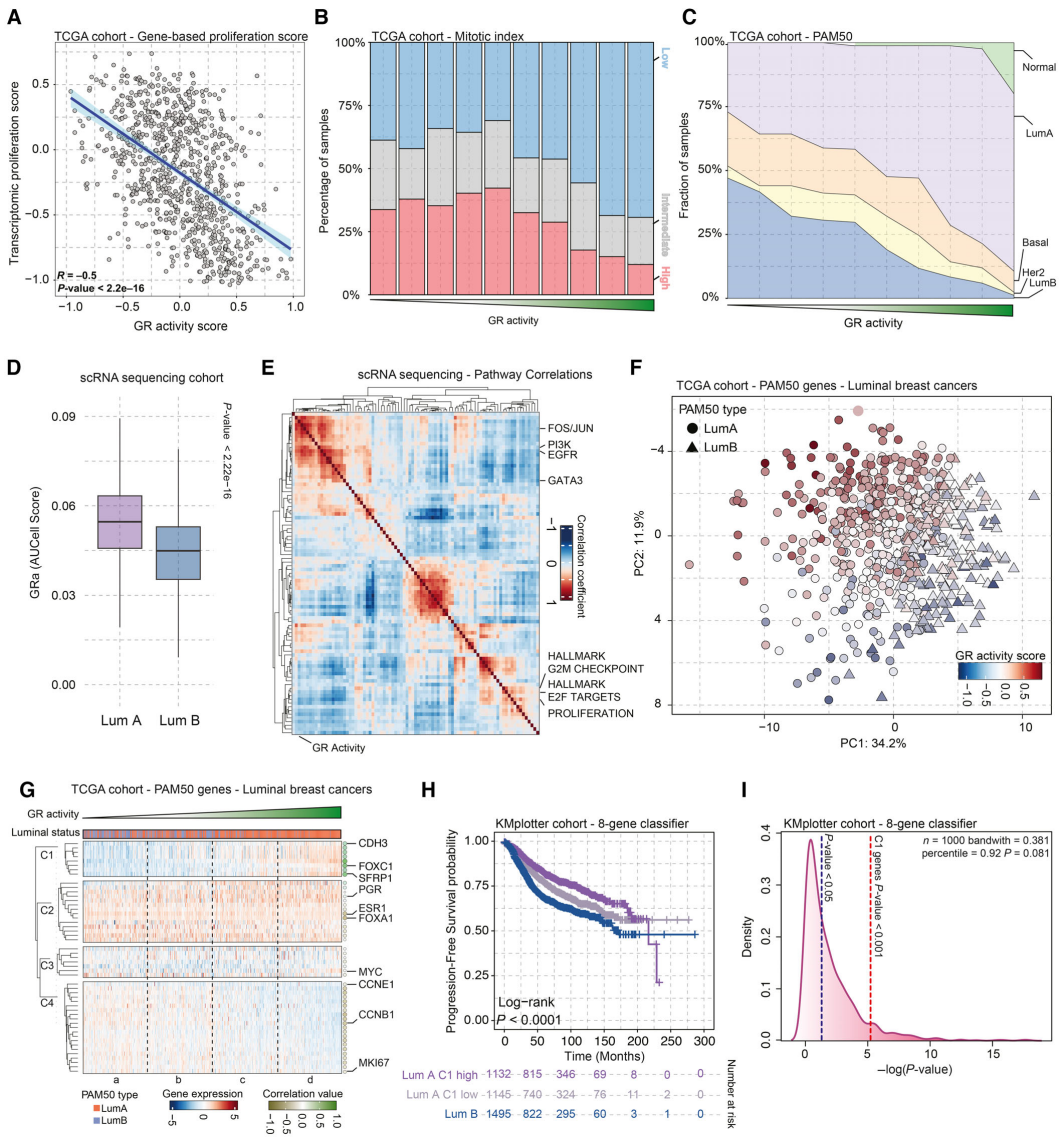
Investigation into the role of GR in human breast cancer Scatterplot depicting the inverse correlation of GR activity signature with transcriptomic cell proliferation gene set in breast cancer samples of the TCGA. *n* = 628.Pathological mitotic index analyses in breast cancer, relative to GR activity signature split in deciles, depicted as percentage of samples with low, intermediate, and high mitotic scores. High GR activity score is associated with low tumor cell mitotic index.Distribution of PAM50 molecular breast cancer subtypes in relation to GR activity levels, in the TCGA cohort. High GR activity is associated with enrichment of luminal A breast cancers, while low GR activity is associated with luminal B tumors. *n* = 850.Single‐cell RNA‐sequencing analyses, exclusively analyzing tumor cells. Higher level of GR activity is found in Luminal A (Lum A; *n* = 7,943) cells than in Luminal B (Lum B; *n* = 4,204) cells. The box begins in the first quartile (25%) and ends in the third (75%), while the line represents the median value. The lines represent segments to furthest data without accounting for outliers. *P*‐values were determined by the Wilcoxon *t*‐test.Pearson correlation heatmap of various pathways including GR activity signature based on data of single‐cell RNA sequencing of cells annotated with Luminal A and B cancer cells. The pathway enrichment scores were calculated using AUCell.PCA analyses of bulk RNA‐sequencing data, focused on PAM50 genes. GR activity separates the ER‐positive breast cancers on PAM50‐based Luminal A (LumA—circle) and B (LumB—triangle) status. *n* = 613.k‐Means clustering analysis of luminal breast cancer samples based on PAM50 genes. *n* = 613.Progression‐free survival probabilities of breast cancer patients (*n* = 3,772) grouped by transcriptomics‐based 8‐gene classifier (Luminal A high expression = purple, Luminal A low expression = gray, and Luminal B = blue). Progression‐free survival probabilities in months are plotted for each group, and censored patients are shown as vertical tick marks.Prognostic power as determined by SigCheck of the 8‐gene classifier (red dotted line) with 1,000 random gene sets of the same size (*P*‐value < 0.05 is indicated by the blue dotted line) for progression‐free survival parameter in KMplot breast cancer cohort (*n* = 3,772).

**Figure EV3:**
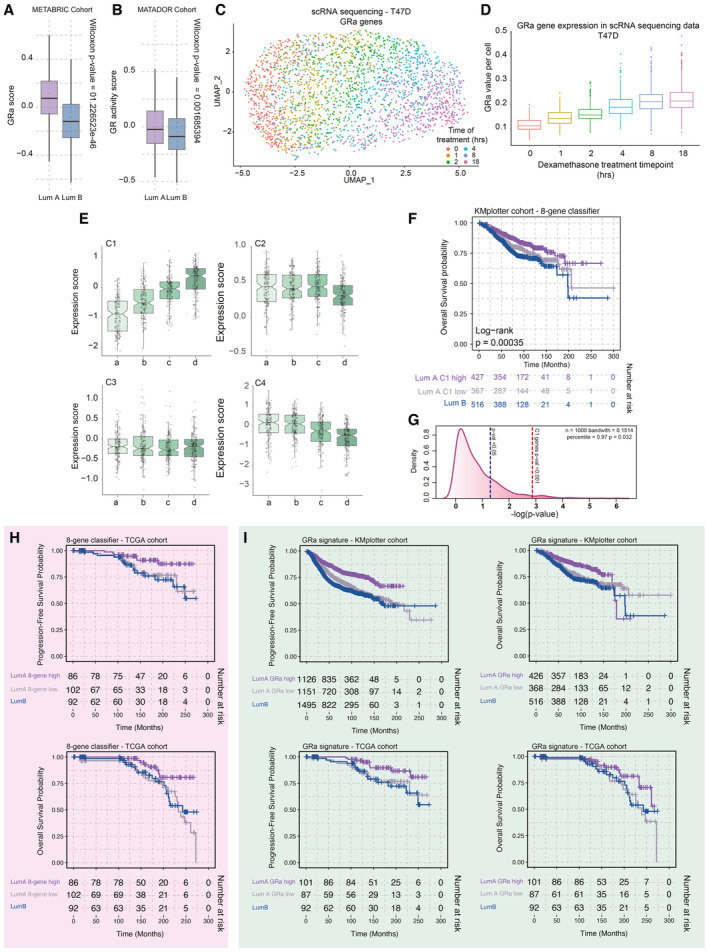
GR activity in breast cancer is linked to luminal subtype status and survival probabilities Boxplots depicting GR activity signature in Luminal A and B cancers of the METABRIC cohort (*n* = 1,134 luminal samples). The box begins in the first quartile (25%) and ends in the third (75%), while the line represents the median value. The lines represent segments to furthest data without accounting for outliers. *P*‐values were determined by the Wilcoxon *t*‐test.Boxplots depicting GR activity signature in Luminal A and B cancers of the MATADOR trial (*n* = 415 luminal samples). *P*‐values were determined by the Wilcoxon *t*‐test. The box begins in the first quartile (25%) and ends in the third (75%), while the line represents the median value. The lines represent segments to furthest data without accounting for outliers.The UMAP analysis of time‐course experiments performed with T47D cell line utilizing the GRa genes. Each cell is colored according to the treatment time points—0, 1, 2, 4, 8, and 18 h.Boxplot depicting the value of GR activity per cell per time point of the time course (400 cells). The central mark indicates the median, and the bottom and top edges of the box indicate the 25^th^ and 75^th^ percentiles, respectively. The lines represent segments to furthest data without accounting for outliers.Average gene expression of genes corresponding to clusters identified by k‐means clustering of PAM50 genes in luminal breast cancer patient samples. The box begins in the first quartile (25%) and ends in the third (75%), while the line represents the median value. The lines represent segments to furthest data without accounting for outliers.Overall survival probabilities of breast cancer patients (*n* = 1,310) grouped by transcriptomics‐based 8‐gene classifier (Luminal A high expression = purple, Luminal A low expression = gray, and Luminal B = blue). Overall survival probabilities in months are plotted for each group, and censored patients are shown as vertical tick marks.Prognostic power as determined by SigCheck of the 8‐gene classifier (red dotted line) with 1,000 random gene sets of the same size (*P*‐value < 0.05 is indicated by the blue dotted line) for overall survival parameter in KMplotter breast cancer cohort (*n* = 1,310).Overall survival probabilities of breast cancer patients (TCGA cohort *n* = 280) grouped by transcriptomics‐based 8‐gene classifier (Luminal A high expression = purple, Luminal A low expression = gray, and Luminal B = blue). Overall survival probabilities in months are plotted for each group, and censored patients are shown as vertical tick marks. First 300 months were included in the analysis.Overall survival probabilities of breast cancer patients (KMplotter cohort *n* = 3,772; TCGA cohort *n* = 280) grouped by GR activity signature (Luminal A high expression = purple, Luminal A low expression = gray, and Luminal B = blue). Overall survival probabilities in months are plotted for each group, and censored patients are shown as vertical tick marks. First 300 months were included in the analysis.

As GR has an active role in breast physiology and development (Tronche *et al*, 1998; Wintermantel *et al*, 2005), we sought to explore and further establish its biological role in breast cancer. Activity of GR correlated negatively with proliferation, both on transcriptional and pathological levels, using mitotic counts as previously determined by expert pathologists (Heng *et al*, 2017) (Fig 2A and B), confirming our above‐mentioned findings. Strikingly, we observed a specific distribution of PAM50 molecular subtypes (Parker *et al*, 2009) of breast cancer in relation to activity of GR (Fig 2C): Samples exhibiting high levels of GRa were mostly luminal A, while most samples with low levels of GRa belonged to the luminal B group (Fig 2C). Higher levels of GR activity in Luminal A samples were also observed in the patients of the METABRIC cohort (Curtis *et al*, 2012) (*n* = 1,134 luminal samples; Fig EV3A), as well as in the NKI in‐house MATADOR trial (Van Rossum *et al*, 2018) (*n* = 415 luminal samples; Fig EV3B), validating our findings across multiple patient series. Considering that GR is ubiquitously expressed among different cell types that jointly contribute to the bulk RNA‐sequencing data, we sought to explore GRa using single‐cell (sc) RNA expression data of human breast cancer. We first interrogated the use of GRa in scRNA‐sequencing data from a time‐course experiment in which T47D breast cancer cell line was exposed to glucocorticoids for up to 18 h (Fig EV3C). This analysis demonstrated that our signature is sensitive and accurate in detecting GR activity in scRNA data (Fig EV3D). Subsequently, we performed GRa calculations in a single‐cell cohort (*n* = 19 luminal samples), on 12,147 malignant breast cancer cells, with a luminal A or luminal B subtype annotation on individual cell basis (Wu *et al*, 2021). We observed that the GRa score is significantly higher in luminal A than luminal B cells (Fig 2D), confirming the observation made on the basis of bulk RNA analysis. Furthermore, in scRNA‐sequencing data, we detected an inverse correlation of GRa with proliferation pathways, as well as a positive correlation with growth receptor signaling pathways (Fig 2E), analogous to our prior findings in lung cancer (Prekovic *et al*, 2021).

To better understand how GR activity relates to luminal breast cancer phenotypes, we performed a PAM50‐based principal component analysis (PCA) of the gene expression TCGA data, focusing on luminal cancers, projecting the GRa scores on top (Fig 2F). This PCA analysis roughly divides the samples into two clusters—one representing, in majority, samples of luminal A cancers and the other consisting of luminal B tumors. Importantly, the GRa score followed this trajectory, with intermingled samples having mostly similar, intermediate‐to‐low, GRa values (Fig 2F). To understand which PAM50 signature genes relate to GRa, we performed a k‐means clustering analysis (Fig 2G). This identified four clusters—C1 (strong correlation; Pearson *R* = 0.659), C2 (high expression and no correlation; Pearson *R* = −0.122), C3 (low expression and no correlation; Pearson *R* = −0.056), and C4 (weak negative correlation; Pearson *R* = −0.375) (Figs 2G and EV3E). Within C4, we found various cell cycle genes that poorly correlate with GRa (e.g., MKI67 ‐ Pearson *R* = −0.217), indicating that the clustering and PCA analysis was not primarily driven by individual cell cycle genes. Cluster C1 containing eight genes (CDH3, KRT17, KRT5, KRT14, EGFR, FOXC1, MIA, and SFRP1) was of particular interest due to its high correlation value with GRa. We proceeded by using the C1 8‐gene classifier in a large cohort (KMplotter composite cohort; Lánczky & Győrffy, 2021) of breast cancers containing transcriptome data and patients' progression‐free survival (PFS; 2,277 luminal A and 1,495 luminal B patients) and overall survival (OS; 794 luminal A and 516 luminal B patients) status. The Kaplan–Meier analysis demonstrated that the C1 8‐gene classifier was able to stratify patients with luminal A cancers into good and poor prognosis groups, on the basis of both PFS and OS parameters (Figs 2H and EV3F). Importantly, the poor prognosis group had a similar survival as patients with luminal B cancers (Figs 2H and EV3F). The C1 8‐gene classifier, derived from the PAM50 gene set, had a highly significant prognostic performance in luminal A patients found in the composite KMplot cohort (Figs 2I and EV3G). These observations were confirmed in the TCGA cohort, for both PFS and OS parameters (Fig EV3H). The same survival analysis was carried out for GRa in both the KMplotter meta‐dataset and TCGA, yielding similar results and demonstrating that GRa is prognostic for patients with luminal A cancers (Fig EV3I).

### Glucocorticoids drive upregulation of the growth‐suppressive *ZBTB16* gene

**Figure 3:**
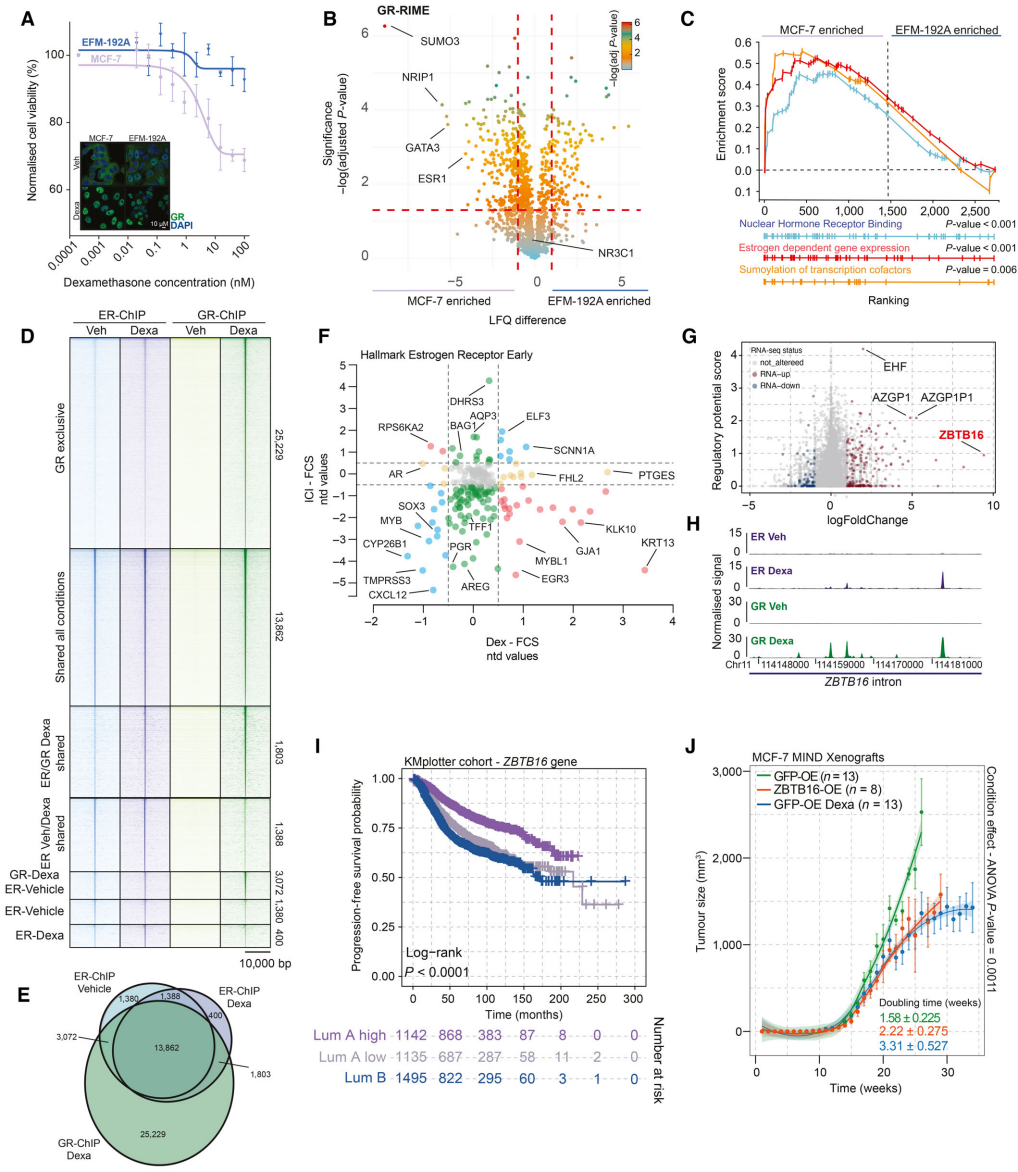
Molecular analysis of GR action in luminal breast cancer models Normalized cancer cell viability in response to glucocorticoid (Dexa) treatment. While EFM‐192A cells (Luminal B) are not affected in their viability following GR activation, MCF‐7 (luminal A) viability was decreased after GR activation, in a dose‐dependent manner. *n* = 4. Mean values ± SEM depicted. In the bottom left corner is a representative immunofluorescence image depicting staining of GR (green) and DAPI (blue) in Luminal A cell line MCF‐7 and Luminal B cell line EFM‐192A. In both cell lines, GR is readily expressed and readily translocated to the nucleus following activation by Dexa. *n* = 3.Volcano plot depicting differentially enriched interactors in GR‐RIME experiments between MCF‐7 and EFM‐192A cell lines. *n* = 3. *P*‐values were determined by two‐sided *t*‐test.Nuclear hormone receptor binding (gene‐set reference: GO:0035257), estrogen‐dependent gene expression (gene‐set reference: R‐HSA‐9018519), and sumoylation of transcription factors (gene‐set reference: R‐HSA‐3232118) GSEA enrichment profiles based on GR‐RIME data comparison between MCF‐7 and EFM‐192 cell lines. *n* = 4.Heatmap of ChIP‐sequencing signal for ER and GR around peak midpoint for all sites detected across the genome in Veh and Dexa conditions. *n* = 3.Venn diagram, depicting shared and unique ChIP‐sequencing‐detected binding sites for ER and GR, in the absence or presence of Dexa. *n* = 3.Effect of ICI (*y*‐axis) and Dexa (*x*‐axis) treatment on expression of ER targets, determined in an RNA‐sequencing experiment. *n* = 3.Regulatory potential analysis of Dexa‐induced ER‐binding sites in relation to gene regulation by Dexa. Genes not altered by Dexa are colored gray, while the up‐ and downregulated genes are depicted as red and blue points, respectively. *n* = 3.Normalized ChIP signal for ER (top, purple) and GR (bottom, green) at ZBTB16 in MCF‐7 cell line, untreated (Veh) or glucocorticoid treated (Dexa). *N* = 3.Progression‐free survival probabilities of breast cancer patients (*n* = 3,772) grouped by ZBTB16 mRNA expression (Luminal A high expression = purple, Luminal A low expression = gray, and LUMINAL B = blue). Progression‐free survival probabilities in months are plotted for each group, and censored patients are shown as vertical tick marks.Normalized tumor growth in xenograft, mammary intraductal models of MCF‐7 cells overexpressing V5‐GFP (green = Veh; blue = Dexa) or V5‐ZBTB16 (orange) in NOD‐SCID‐γ mice. Arrows indicate when treatment was initiated for the Dexa‐treated group. Mean values ± SEM depicted (GFP Veh *n* = 13, GFP Dexa *n* = 13, and ZBTB16 *n* = 8 animals). Condition effect *P*‐value was determined by mixed‐model ANOVA (Tukey's multiple‐comparison test). Data information: All experiments were performed in biological replicates and the number (*n*) of replicates indicated. Source data are available online for this figure.

**Figure EV4:**
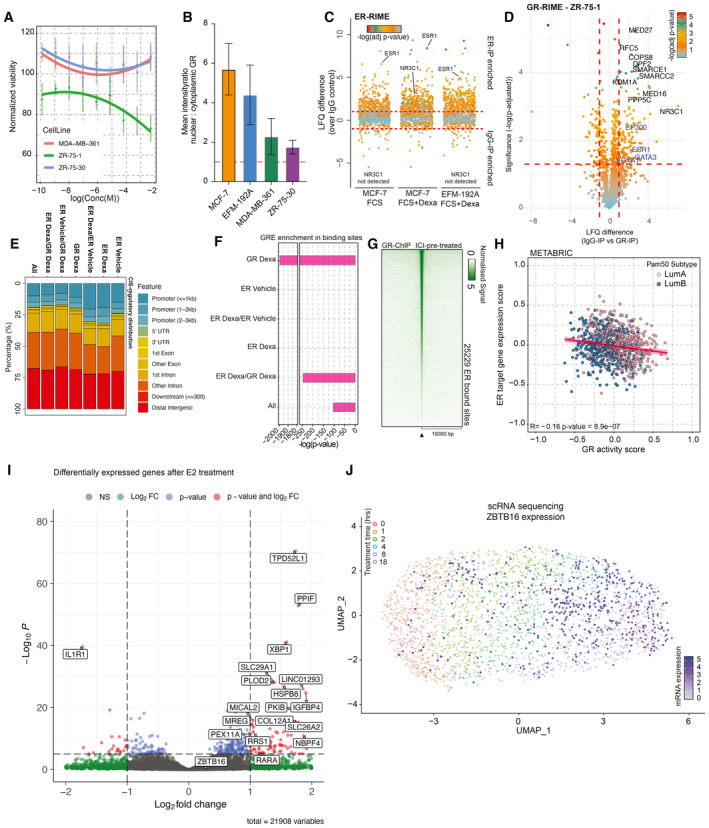
Molecular aspect of GR signaling in luminal breast cancer Normalized cancer cell viability (lines depict polynomial fit) in response to GR agonist (Dexa) treatment. *n* = 4. Mean values ± SEM depicted.Bar plot depicting nuclear‐to‐cytoplasmic ratio of GR in various cell lines. *n* = 3. Mean values ± SEM depicted.Jitter plot depicting differentially enriched interactors in ER‐RIME experiments between Veh‐treated MCF‐7 and Dexa‐treated MCF‐7 and EFM‐192A cell lines. *n* = 4. *P*‐values were determined by two‐sided *t*‐test.Volcano plot depicting enriched (in comparison to IgG‐IP control) interactors in GR‐RIME experiments in ZR‐75‐1 cell line. *n* = 3. *P*‐values were determined by two‐sided *t*‐test.Genomic distribution analyses for all the sites detected in ChIP‐sequencing experiments. *n* = 3.HOMER motif analysis of *P*‐value for GREs across different categories of sites.Heatmap of ChIP‐sequencing signal for GR around peak midpoint for all sites detected across the genome after 24 h pre‐treatment with ICI and subsequent 2 h treatment with Dexa. *n* = 3.Scatterplot depicting the absence of correlation of GR activity with ER target gene expression in luminal breast cancers.Volcano plot depicting transcriptomic differences between DCC‐treated and DCC+E2‐treated MCF‐7 (*n* = 4). Adjusted *P*‐values were determined by DESeq2 (Wald test *P*‐values corrected for multiple testing using Benjamini and Hochberg method).The UMAP analysis of time‐course experiments performed with T47D cell line utilizing the GRa genes. Each cell is colored according to the treatment time points—0, 1, 2, 4, 8, and 18 h, and the values of *ZBTB16* gene expression are projected on top (white‐to‐blue gradient). Data information: All experiments were performed in biological replicate and the number (*n*) of replicates indicated.

The mechanism through which glucocorticoids affect luminal breast cancer biology is unclear. Therefore, we explored the role of GR activity in *in vitro* models, derived from luminal A and luminal B cancers. Dexa, a synthetic glucocorticoid, reduced viability in luminal A tumor‐derived models (MCF‐7 and ZR‐75‐1), but had no detectable effect on viability of luminal B tumor‐derived cell lines (EFM‐192A, MDA‐MB‐361, and ZR‐75‐30) (Figs 3A and EV4A). By means of confocal microscopy, we showed that insensitivity to GR‐agonist treatment may be due to diminished GR nuclear translocation for several of these cell lines (Fig EV4B; MDA‐MB‐361 and ZR‐75‐30). Therefore, for subsequent experiments, we focused on the EFM‐192A cell line, which exhibited nuclear translocation of GR, similar to models derived from luminal A cancers (Fig EV4B), yet without exposing a growth inhibitory response to GR stimulation. As GR recruits interactors necessary for its function as a transcription factor (Vandevyver *et al*, 2014; Kulik *et al*, 2021), we profiled the GR protein complex by means of rapid immunoprecipitation mass spectrometry of endogenous proteins (RIME; Mohammed *et al*, 2013). Strikingly, we identified that the GR complex in the MCF‐7 luminal A cancer‐derived cell line is significantly enriched for the estrogen receptor protein (Fig 3B) and its complex members (Fig 3C), in comparison to the GR complex in the luminal B cancer‐derived cell line, EFM‐192A. We confirmed these findings by performing ER‐RIME in MCF‐7 and EFM‐192A cells, where GR was observed in the ER complex only in MCF‐7 cells exposed to Dexa (Fig EV4C). The observation of GR‐ER interactions extends beyond MCF‐7 cells, as demonstrated by GR‐RIME experiments in dexamethasone‐treated ZR‐75‐1 cells (Fig EV4D). To further characterize the cross‐talk between GR and ER, we performed ChIP‐sequencing analysis for both factors under Veh and Dexa conditions in the MCF‐7 cell line that was cultured in full media containing estradiol (Fig 3D). Following peak calling using MACS (Zhang *et al*, 2008), and consensus replicate analysis using MSPC (Jalili *et al*, 2015), we performed peak overlap evaluation (Fig 3E). The majority of ER‐binding sites detected in both Veh and Dexa conditions were also occupied by GR, while we detected a large number of sites exclusively occupied by GR (Fig 3D and E). Next, we annotated the peaks using CEAS analysis (Ji *et al*, 2006), which showed expected genomic distribution of the binding sites for both GR and ER, across different conditions (Fig EV4E). Importantly, using HOMER motif analysis (Heinz *et al*, 2010), glucocorticoid‐binding motifs (GRE) were detected in ER/GR co‐occupied regions (Fig EV4F), suggesting that GR/ER co‐binding does not occur via tethering, contrasting prior findings (Yang *et al*, 2017). This is further supported by ChIP‐sequencing experiments in which we pre‐treated MCF‐7 cells with Fulvestrant (ICI), a selective ER degrader (Osborne *et al*, 2004), for 1 day and then exposed them to Dexa. Despite ER degradation, Dexa‐activated GR was still capable of occupying previously identified ER co‐bound regions (Fig EV4G), arguing against a tethering mode of action.

As strong overlap of GR and ER binding exists, we explored if there is any modulation of ER target gene expression following GR activation. Treatment with ER degrader ICI diminished the expression of the ER target genes (Liberzon *et al*, 2015) (gene‐set reference: M5906), while Dexa did not have a major effect on expression of most of ER‐target genes (Fig 3F). These data suggest that GR may only have a weak modulatory effect on ER action, despite high degree of binding site overlap. This conclusion is further supported by analysis of tumor samples where we did not detect significant correlation between GRa and ER signatures (Fig EV4H).

We next focused on genomic regions to which we detected ER binding solely upon GR activation (Fig 3E; *n* = 1,803 sites). Utilizing Cistrome‐GO (Li *et al*, 2019), we functionally annotated these sites to individual genes, yielding a score that represents the likelihood of regulation (i.e., regulatory potential (RP)). By relating the RP to differential expression of genes following Dexa treatment, we identified *ZBTB16* gene as a potential driver of Dexa‐induced growth suppression (Fig 3G). As seen from the binding profiles, ER is actively occupying the *ZBTB16* intron sites only once GR is activated and bound at overlapping locations (Fig 3H). Upregulation of *ZBTB16* expression was not observed when activating ER in the absence of glucocorticoid (Fig EV4I). Whether ER transcriptional activity is necessary for upregulation of *ZBTB16* expression, or whether ER only acts as modulator of GR‐driven upregulation of *ZBTB16*, remains unknown and should be addressed by future studies. Importantly, the glucocorticoid‐induced upregulation of *ZBTB16* is not exclusive for MCF‐7, and is also seen in T47D cells; another model derived from a luminal A cancer (scRNA‐sequencing time course; Fig EV4J).

**Figure EV5:**
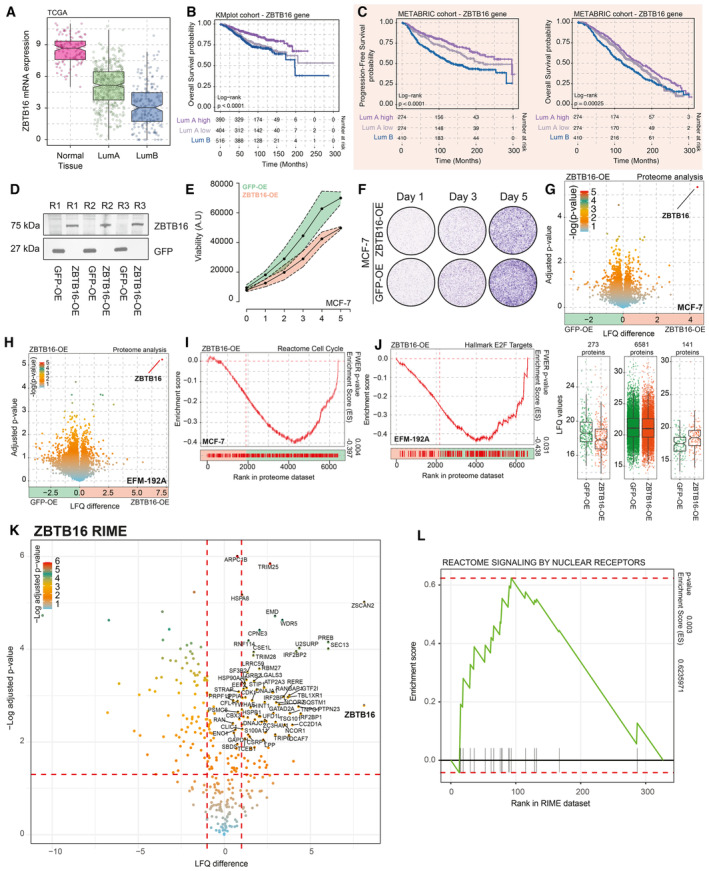
*ZBTB16* action in ER‐positive breast cancer Boxplot depicting *ZBTB16* mRNA expression in normal breast tissues, Luminal A, as well as Luminal B cancer samples (TCGA cohort *n* = 702). The box begins in the first quartile (25%) and ends in the third (75%), while the line represents the median value. The lines represent segments to furthest data without accounting for outliers.Overall survival probabilities of breast cancer patients (KMplotter meta dataset; *n* = 1,310) grouped by *ZBTB16* mRNA expression (Luminal A high expression = purple, Luminal A low expression = gray, and Luminal B = blue). Overall survival probabilities in months are plotted for each group, and censored patients are shown as vertical tick marks.Progression‐free and overall survival probabilities of breast cancer patients (METABRIC dataset) grouped by *ZBTB16* mRNA expression (Luminal A high expression = purple, Luminal A low expression = gray, and Luminal B = blue). Progression‐free and overall survival probabilities in months are plotted for each group, censored patients are shown as vertical tick marks.Western blot showing expression of V‐5‐tagged GFP and ZBTB16. *n* = 3.Normalized cancer cell viability for MCF‐7 GFP (green) and ZBTB16 (orange) overexpression models. *n* = 4.Representative crystal violet assay image for MCF‐7 GFP and ZBTB16 overexpression models. *n* = 3.Upper panel, volcano plot depicting differences in protein expression between GFP‐OE and ZBTB16‐OE models in MCF‐7 cells. Significance is depicted as a color gradient; lower panel, intensity (LFQ) values per category (decrease in expression, same levels, and increase in expression upon ZBTB16 overexpression). *n* = 4. The box begins in the first quartile (25%) and ends in the third (75%), while the line represents the median value. The lines represent segments to furthest data without accounting for outliers.Volcano plot depicting differences in protein expression between GFP‐OE and ZBTB16‐OE models in EFM‐192A cells. *n* = 4.Reactome cell cycle (gene‐set reference: R‐HSA‐1640170) GSEA enrichment profiles based on whole‐proteome data comparison between GFP‐OE and ZBTB16‐PE in MCF‐7 cell line.Hallmark E2F targets (gene‐set reference: M5925) GSEA enrichment profiles based on whole‐proteome data comparison between GFP‐OE and ZBTB16‐PE in EFM‐192A cell line.Volcano plot depicting differentially enriched (over IgG control) interactors in ZBTB16‐RIME experiments in MCF‐7 cells. *n* = 4. *P*‐values were determined by two‐sided *t*‐test.GSEA enrichment profiles for “Reactome Signaling by nuclear receptors” gene sets based on IgG versus ZBTB‐16 RIME comparison (*n* = 4). Data information: All experiments were performed in biological replicates and the number (*n*) of replicates indicated.

As higher mRNA expression of the transcriptional repressor *ZBTB16* (Han *et al*, 2019) is observed in normal breast tissue in comparison to primary tissue, with further decrease in luminal B cancers (Fig EV5A), we sought to further understand the role of *ZBTB16* in ER‐positive breast cancer. Importantly, expression of *ZBTB16* itself was predictive of survival in patients with luminal A cancer, with high expression relating to favorable PFS and low expression to poor PFS, the latter with comparable outcomes as observed for luminal B subtype (Fig 3I). This was also observed for OS in the same cohort (Fig EV5B), and validated in the METABRIC population (Fig EV5C). To functionally study *ZBTB16*, we overexpressed a V5‐tagged version of the protein (Fig EV5D) which dampened proliferation dynamics in MCF‐7 cells, compared to V5‐tagged GFP control (Fig EV5E and F). Furthermore, overexpression of *ZBTB16* led to a high level of gene repression in both MCF‐7 and EFM‐192A models, as seen in whole proteome analysis (Fig EV5G and H). In both cell lines, we observed a significant repression of genes related to proliferation (Fig EV5I and J). With ZBTB16 having established role in epigenetic regulation (Barna *et al*, 2002), we sought to learn more about its interaction network and performed RIME experiments in the MCF‐7 cell line overexpressing ZBTB16. This revealed the interactome of ZBTB16 which includes various proteins involved in transcriptional regulation (e.g., GATAD2A and STAT3) and the ubiquitin–proteasome system (e.g., TRIM25 and UFD1L). Of particular interest is the interaction with numerous nuclear receptor partners (Fig EV5K and L), such as NCOR proteins that play a role in recruitment of histone deacetylases (HDACs), which is in line with the previously reported function of ZBTB16 as a transcriptional repressor (Barna *et al*, 2002).

Importantly, we used NOD‐SCID‐γ mice and injected MCF‐7 cells overexpressing either GFP (GFP‐OE, control) or *ZBTB16* (ZBTB16‐OE) into their mammary ducts using the MIND model procedure (Sflomos *et al*, 2016). After the total tumor size reached 100 mm^3^, we randomized the GFP‐OE group in two arms—one receiving Veh and the other receiving Dexa (4 mg/kg) treatment. The xenograft growth curves demonstrate that overexpression of *ZBTB16* (ZBTB16‐OE) led to diminished growth rates in comparison to the GFP‐OE counterparts, comparable to the slower growth dynamics caused by Dexa treatment (Fig 3J; doubling time in weeks: GFP‐OE = 1.58 ± 0.225; ZBTB16‐OE = 2.22 ± 0.275; and GFP‐OE Dexa = 3.31 ± 0.527). The *in vitro*, *in vivo*, and clinical data position *ZBTB16* as an important hormone‐driven factor that diminishes luminal breast cancer growth and progression.

### Inhibiting BRDs and HDACs diminishes viability of breast cancers without GR activity

**Figure 4:**
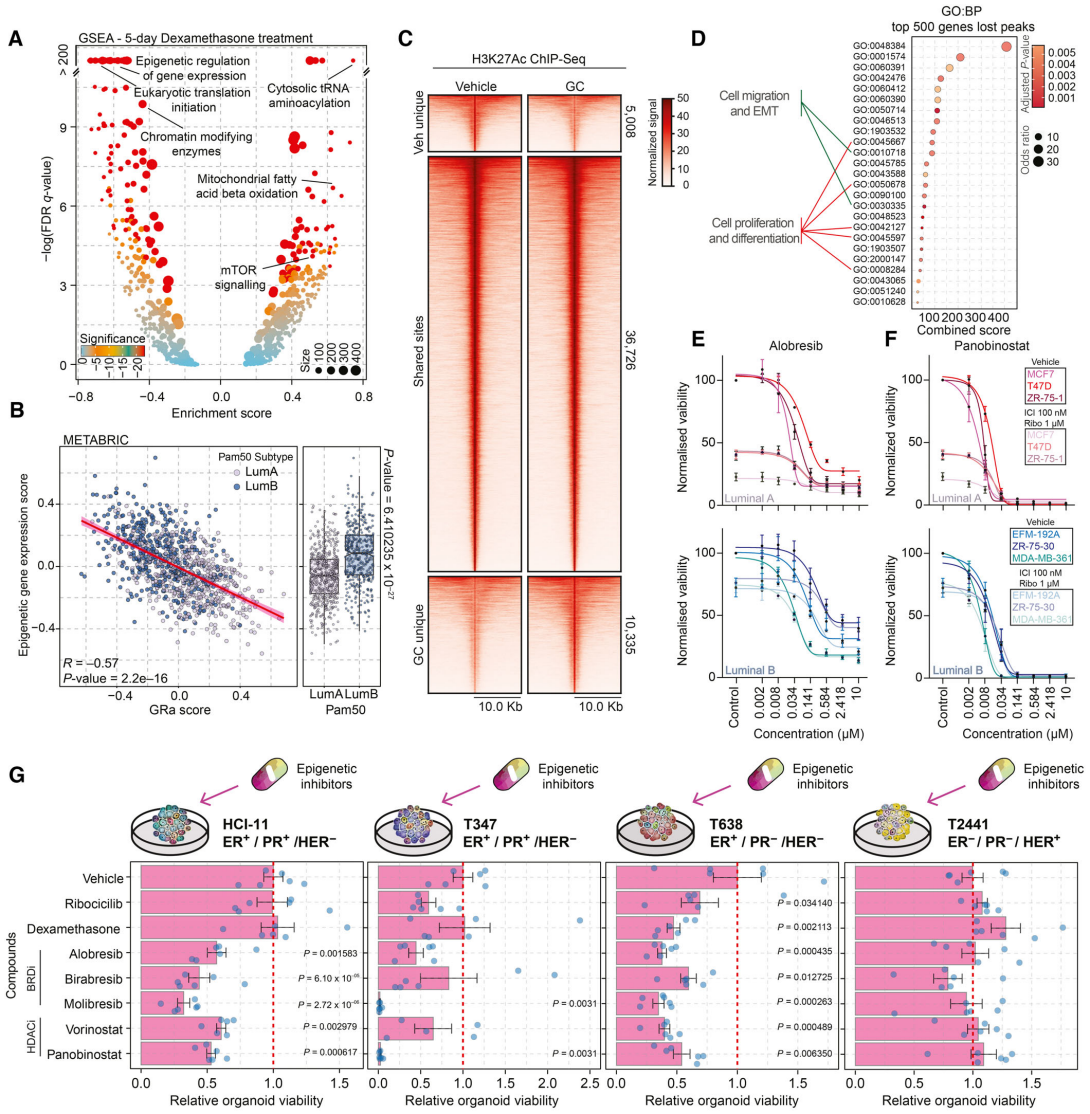
Examining the GR signaling to leverage discovery of new therapeutic strategies Volcano plot depicting GSEA results of whole‐proteome data corresponding to 5‐day Dexa treatment of MCF‐7 cells. Significance is depicted as a color gradient, and the size of the dots represents the size of the gene set. *n* = 4.Scatterplot showing correlation of GR activity signature with the mean expression of various epigenetic regulators (gene‐set reference: R‐HSA‐212165) in luminal breast cancer samples of the METABRIC cohort. *n* = 958. Pearson *R‐* and *P*‐value are reported.Heatmap of H3K27Ac ChIP‐seq signal in MCF‐7 cells treated with vehicle or Dexa for 7 days. Data are centered at H3K27Ac peaks, depicting a ±10 kb window around the peak center. Data represent the average of three biological replicates.Pathway overrepresentation analysis (GO:BP) for the top 500 genes found to be potentially regulated by the vehicle unique H3K27Ac peaks. Significant pathways are depicted, and pathways involved in cell migration and EMT as well as cell proliferation and differentiation are highlighted.Normalized cancer cell viability of Luminal A (MCF‐7, T47D, and ZR‐75‐1) and Luminal B (EFM‐192A, ZR‐75‐30, and MDA‐MB‐361) cell lines in response to BRD inhibitor (Alobresib) treatment. Treatment lasted for 5 days. *n* = 4. Lines (sigmoidal fit) and mean value ± SEM depicted.Normalized cancer cell viability of Luminal A (MCF‐7, T47D, and ZR‐75‐1) and Luminal B (EFM‐192A, ZR‐75‐30, and MDA‐MB‐361) cell lines in response to HDAC inhibitor (Panobinostat) treatment. Treatment lasted for 5 days. *n* = 4. Lines (sigmoidal fit) and mean value ± SEM depicted.Bar chart showing response to various compounds for four breast cancer organoid lines (HCI‐11, T347, T638, and T2441) alongside key tumor characteristics displayed on top of the graphs. Drug concentrations (dexamethasone 100 nM, ribociclib 1,000 nM, alobresib 100 nM, birabresib 500 nM, vorinostat 500 nM, panobinostat 10 nM, and molibresib 500 nM). *n* = 6. Error bars represent mean ± SEM. *P*‐values were determined by Dunnett's test and only displayed if under the *P*‐value of 0.05 cutoff. Data information: All experiments were performed in biological replicates and the number (*n*) of replicates indicated. Source data are available online for this figure.

**Figure EV6:**
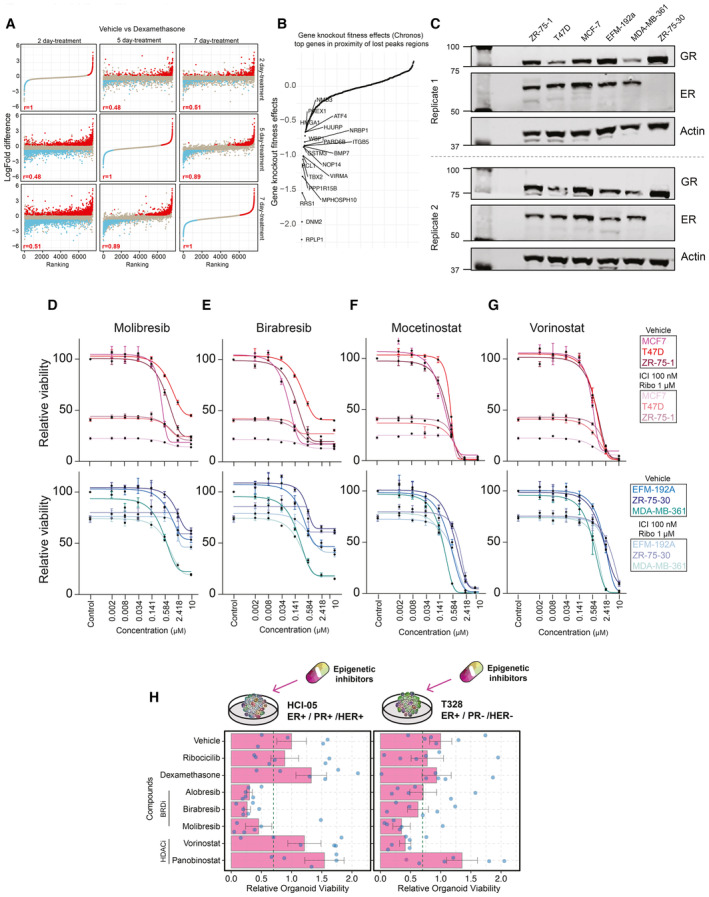
Inhibition of epigenetic pathways in additional models of breast cancer Comparison of Dexa‐induced proteomic changes in proteomics experiments across different time points (2‐ vs. 5‐ vs. 7‐day treatment). *n* = 4. Adjusted *P*‐values (*P*adj) were determined by *t*‐test (*P*‐values corrected for multiple testing using Benjamini and Hochberg method). Pearson correlation value is reported.Snake plot depicting the gene knockout fitness effects (Chronos) for top genes potentially regulated by vehicle unique H3K27Ac sites.Western blot showing expression of GR and ER in all the breast cancer cell line models used in the manuscript, with actin as a loading control (*n* = 2).Normalized cancer cell viability in response to BRD inhibitor (Molibresib) treatment. *n* = 4.Normalized cancer cell viability in response to BRD inhibitor (Birabresib) treatment. *n* = 4.Normalized cancer cell viability in response to HDAC inhibitor (Mocetinostat) treatment. *n* = 4.Normalized cancer cell viability in response to HDAC inhibitor (Vorinostat) treatment. *n* = 4.Bar chart showing response to various inhibitors for each of the organoid lines alongside key tumor characteristics. Drug concentrations (dexamethasone 100 nM, ribociclib 1,000 nM, alobresib 100 nM, birabresib 500 nM, vorinostat 500 nM, panobinostat 10 nM, and molibresib 500 nM). Error bars represent mean ± SEM. *n* = 6. Data information: All experiments were performed in biological replicates and the number (*n*) of replicates indicated.

Despite advancements and various treatment options offered for patients with luminal A or B cancers, the survival of patients with aggressive cancer is limited. While *ZBTB16* may be important for the growth arrest phenotype, this cannot be therapeutically exploited yet. Therefore, to discover new therapeutic routes, we have studied which pathways are repressed by GR activation on proteomic level. We performed Dexa treatment time‐course proteomic experiments in MCF‐7 cell line, and observed that a stable effect on the proteome level was achieved after 5‐day exposure in culture (Fig EV6A). The gene‐set enrichment analysis of proteomics data revealed that GR represses various pathways, including those related to epigenome regulation (Fig 4A). Importantly, the negative relation between epigenome regulation gene sets and GR was also observed in clinical samples, where GRa inversely correlates with their expression (Fig 4B, *R* = −0.57; gene‐set reference: R‐HSA‐212165). As others linked glucocorticoids to epigenetic changes (Mourtzi *et al*, 2021), we explored if GR activation alters the H3K27Ac landscape, related to active enhancers, of MCF‐7 cells by performing ChIP‐seq. After a long‐term glucocorticoid exposure of 7 days, we observed an extensive alteration in H3K27Ac profiles (Fig 4C). Of particular interest are the sites that lose H3K27Ac, as these are found to potentially regulate genes involved in cell migration and EMT as well as cell proliferation and differentiation as determined by the overrepresentation analysis using the gene ontology: biological processes pathways (Fig 4D). Importantly, using the CRISPR screen data from MCF‐7 cells available through the DepMap portal (https://depmap.org), we observed that various genes are essential for cell survival (Fig EV6B). The loss of H3K27Ac at specific sites following exposure to glucocorticoids may be related to repressive functions of ZBTB16, which may potentially act via interaction with NCOR proteins and recruitment of HDACs, as suggested by our RIME experiments. While our efforts to perform ChIP‐sequencing analyses of endogenous ZBTB16 following GR activation were not successful, in line with prior reports (Mao *et al*, 2016), future studies should explore if ZBTB16 is directly involved in glucocorticoid‐induced loss of histone acetylation.

In relation to the above‐mentioned findings, we sought to explore whether epigenetic inhibitors would be of use to treat luminal breast cancers. To test if epigenetic inhibitors would outperform standard‐of‐care treatment (Fulvestrant (“ICI”) and Ribociclib), we tested a panel of drugs on luminal A‐derived (MCF‐7, T47D, and ZR‐75‐1) and luminal B‐derived (EFM‐192A, ZR‐75‐30, and MDA‐MB‐361) models. The models used showed no major differences in expression levels of GR and ER (Fig EV6C), except the ZR‐75‐30 model that had undetectable levels of ER (in line with previously published observations; Shyu *et al*, 2005). The cells were treated with different concentrations of BRD (alobresib, molibresib, and birabresib) and HDAC (panobinostat, mocetinostat, and vorinostat) inhibitors in combination with standard of care or as single agent for 5 days. Each of the BRD and HDAC inhibitors diminished breast cancer cell viability within a desirable molar range (0.008–0.5 μM), and led to further reduction in viability across all models tested when combined with standard of care (Fig 4E and F). Specifically, models derived from luminal B cancer appear not to respond well to standard‐of‐care treatment (Fig 4E and F); an observation consistent with recently reported findings in small‐size clinical cohorts (Turner *et al*, 2019; Shao *et al*, 2021). In this case, addition of epigenetic inhibitors to the treatment regimen provided a significant reduction in cancer cell viability (Figs 4E and F, and EV6D–G). Importantly, using five luminal organoid models and one non‐luminal control organoid (ER^−^/PR^−^/HER2^+^) derived from breast cancer (Cosgrove *et al*, 2022; Guillen *et al*, 2022), we successfully validated our cell line‐based observations (Figs 4G and EV6H). Jointly, these data suggested that mimicking GR pathway repression through epigenetic inhibitors may enhance efficacy of current treatment regiments to improve survival rates in aggressive luminal ER^+^ breast cancer.

## Discussion

Breast cancer is a complex and heterogeneous disease, encompassing various molecular subtypes, with the luminal subtype being the most‐predominant one (Perou *et al*, 2000). All luminal tumors are characterized by the expression of ER; however, luminal B tumors exhibit increased expression of proliferative and/or cell‐cycle genes compared to luminal A tumors leading to worse prognosis (Prat *et al*, 2015). The biological foundations of luminal disease remain enigmatic, justifying the need to identify novel biomarkers and therapeutic targets for patients with challenging subtypes.

Our study into the role of GR in primary cancers, particularly breast cancer, was aimed to better explain its role in cancer biology, and particularly in relation to cancer growth. We propose that loss of GR activity constitutes the driving force behind progression of luminal breast cancers. In relation to loss of GR activity, prior research has shown that GR may be inactivated through diverse mechanisms, including alterations in nuclear translocation capacity (Matthews *et al*, 2004) and co‐regulator recruitment (Prekovic *et al*, 2021). In the context of breast cancer, *NR3C1* promotor methylation (Nesset *et al*, 2014) has been suggested to play a role in its transcriptional silencing; however, other possible mechanisms of inactivation may also exist and should be dissected in further studies. Nonetheless, loss of GR action seems to be intricately associated with the uncoupling of GR from ER. The cross‐talk between nuclear receptors may be one of the leading mechanisms that control proliferative capacity of breast cancer (Mayayo‐Peralta *et al*, 2021a), However, it remains unclear how GR and ER interactions occur. It was previously suggested that co‐occupancy of these nuclear receptors is due to tethering of GR to DNA‐bound ER (Yang *et al*, 2017), resulting in repression of the ER transcriptional program. Studies on human breast cancers (Severson *et al*, 2018), as well as our data, do not support this but rather suggest that GR binds in proximity of ER‐binding sites possibly competing with its binding to DNA. Future research should address the mechanistic underpinnings of GR‐ER cross‐talk, and particularly focus on how ER action is altered by GR activation. Importantly, as we show here, loss of GR activity leads to the absence of *ZBTB16* expression—a crucial hormone‐driven regulator of cancer growth. Intriguingly, *ZBTB16* gene expression appears to be governed by multiple nuclear receptors, such as the androgen receptor (Hickey *et al*, 2021). This multifaceted regulation positions *ZBTB16* as an important suppressor of tumorigenesis, underscoring its potential significance in maintaining cellular homeostasis and cancer growth regulation. This is supported by studies in other cancer types where *ZBTB16* has been associated with growth arrest and diminished cancer growth (Wang *et al*, 2013; He *et al*, 2020). Our study offers an insight into the fundamental mechanisms contributing to the diversity of breast cancer subtypes and accentuates the critical role of GR in reducing the proliferative capacity of luminal breast cancer.

Considering the heterogeneity of breast cancer subtypes, GR has been implicated in driving the progression and metastasis of ER‐negative disease (West *et al*, 2018; Obradović *et al*, 2019), which accounts for approximately 15% of all breast cancers (Colleoni *et al*, 2016; Esserman *et al*, 2017; Pan *et al*, 2017; Lindström *et al*, 2018; Nancy *et al*, 2019). The precise mechanisms underlying the contrasting roles of GR in ER‐negative and ER‐positive disease remain to be fully elucidated but may involve factors such as *ZBTB16* regulation, as well as variations in co‐regulator recruitment, DNA binding profiles, or potential interaction with other signaling pathways. It is imperative to acknowledge that GR can exert distinct effects in various subtypes of breast cancer, and this information should be incorporated when devising clinical strategies for patients.

As we show here, activation of GR leads to changes in the H3K27Ac landscape of breast cancer cells. Of particular importance, glucocorticoid exposure leads to loss of H3K27Ac in vicinity of genes involved in proliferation and metastasis. Conversely, these sites are marked by H3K27Ac when GR is inactive and are potentially of high importance in progression of breast cancer. Inspired by these findings, we used various epigenetic inhibitors (that block action of either HDACs or BRDs) to position these as promising therapeutic strategies for cancers that do not have an active GR‐signaling axis, such as luminal B breast cancers. Epigenetic inhibitors are utilized to treat hematological malignancies but their single‐agent efficacy is yet to be established in solid cancers. The limited success of these treatments in solid cancers can potentially be attributed to patient group selection. Therefore, future studies in breast cancer, especially in luminal subtypes, may not only use GR activity as a biomarker but also utilize the new generation of epigenetic inhibitors, applied alone or in combination with standard‐of‐care therapeutics for breast cancer.

In conclusion, our study offers novel insights into the significance of GR signaling in modulating breast cancer's proliferative capacity and presents potential pathways for clinical translation of epigenetic‐based therapies to benefit breast cancer patients. Notably, we have developed a molecular signature to determine GR activity, which can be utilized across various models, enabling clinicians to stratify patients and tailor therapies accordingly. The identification of biomarkers and therapeutic targets correlated with GR signaling may have direct implications for developing personalized treatment strategies for breast cancer patients based on their unique molecular subtypes. Ultimately, these discoveries may accelerate the development of more effective targeted therapies and enhanced clinical outcomes for breast cancer patients.

## Materials and Methods

### Cell lines

MCF‐7, ZR‐75‐1, T47D, ZR‐75‐30, EFM‐192A, MDA‐MB‐361, and HEK293T cells were obtained from the American Type Culture Collection (ATCC). All cells were kept at 37°C with 5% CO_2_. MCF‐7, ZR‐75‐1, HEK293T, and ZR‐75‐30 cells were cultured in Dulbecco's modified Eagle medium (DMEM, Life Technologies) supplemented with 10% fetal calf serum (FCS). EFM‐192A and MDA‐MB‐361 cells were maintained in DMEM supplemented with 20% FCS. All media contained 1% penicillin–streptomycin (5,000 U/ml). All cell lines were authenticated by STR profiling and tested negative for mycoplasma throughout the course of the project.

### Compounds

The following drugs were used in this study: dexamethasone (HY‐14648), fulvestrant (HY‐13636), ribociclib (HY‐15777), alobresib (HY‐109050), molibresib (HY‐13032), birabresib (HY‐15743), vorinostat (HY‐10221), panobinostat (HY‐10224), and mocetinostat (HY‐12164). All of these drugs were purchased from MedChemExpress.

### Overexpression cell line generation

Overexpression plasmids in a plx‐304 backbone, containing V5‐tagged GFP and ZBTB16 were obtained from the Jacqueline Jacobs lab (NKI) and Broad ORF library (Yang *et al*, 2011), respectively. Overexpression vectors were co‐expressed with third‐generation viral vectors in HEK293T cells using polyethyleneimine (PEI). After lentivirus production, the medium was harvested and transferred to the designated cell lines. Two days post‐infection, cells were put on blasticidin selection for 10 days.

### GR activity signature development

Initially, 55 datasets were assembled (Table EV1), containing transcriptomics data of various cell models, treated with vehicle or glucocorticoids (type indicated in Table EV1). As multiple‐sequencing technologies were used, each dataset was initially analyzed separately. Of all the datasets collected, 22 datasets were discarded due to technical issues. In some of the cases, the data were available in processed form only, therefore the filtering could not be redone and performed according to our standards. Moreover, a few experiments contained only one replicate, therefore differential expression analysis could not be executed. In total, for the final analysis, we included 12 datasets generated using RNA‐sequencing technology (Illumina) with nine of these being in‐house experiments and another 26 datasets were produced using microarray (Affymetrix and Agilent Technologies) analysis. To process the data, distinctive scripts were implemented in *R* for handling each individual dataset using DESeq2 for RNA‐sequencing data and limma for microarray sequencing.

Dexamethasone was used in 26 experiments, prednisone was the glucocorticoid of choice in two experiments, and hydrocortisone was used in nine cases. After gene duplicate filtering, the data were fit to the model, to estimate the expression levels for each assigned group (control and GCs treatment). Then, the empirical Bayes step is applied to get differential expression statistics (*t*‐test and *F*‐statistic) and *P*‐values by moderation of the standard error toward a global value. Finally, all differentially expressed genes were extracted after matching the identification probe with their gene name in a list format for further process.

For the 33 datasets remaining, we filtered the genes on basis of log_2_ fold change (logFC; cutoff > 0.5) and (adjusted) *P*‐value (cutoff < 0.05). Due to unusually high number of genes, upregulated five datasets were removed. Subsequently, only upregulated genes were selected mimicking the design of Hallmark activity gene sets for other nuclear receptors—ER and AR (Liberzon *et al*, 2015). Genes were then filtered on the basis of their occurrence in the datasets. We opted for genes that are represented in at least three models. Furthermore, we downsized the gene set using the TCGA mRNA expression dataset, selecting only the genes that positively correlate with *NR3C1* mRNA levels (*R* > 0). For calculations of GR activity, gene expression was first transformed to a *z*‐score (equation 1) and then averaged per patient, therefore the mean of those genes could be represented as a single value, which will be addressed as GR activity from now on.

(1) $$z‐\text{score}=\frac{\left(\text{score}-\text{mean}\right)}{\mathrm{std}}$$

The association of GR activity score with *NR3C1* mRNA levels was evaluated per patient and equation (2) was used to fit the data.

(2) $$\mathrm{lm}\left(\mathrm{GR}\;\text{activity}\right)∼\text{poly}\left(\text{patients},\text{degree}\right)$$

It displays a linear model (*lm*) that uses polynomial (*poly*) regression to fit the model. The degree that was used was 6, since after that point the difference is minimal. The trend was calculated by using the *R* ^2^.

(3) $$R^{2}=1-\frac{\mathrm{SSE}}{\mathrm{SST}}$$

*R* ^2^ is the proportion of variation in the dependent variable. *SSE* is the sum of squared errors and *SST* is the sum of squared total. It was used as a metric to compare GR activity score signature against mRNA levels with other gene sets of equal number of genes.

### TCGA (Pan‐Cancer) ssGSEA dataset

For the purpose of pathway analysis, the TCGA single‐sample gene set enrichment analysis (ssGSEA) dataset was used (Charles Vaske and Steve Benz named Pathway Representation and Analysis by Direct Reference on Graphical Models (PARADIGM; Vaske *et al*, 2010)), which combines the pathway, expression, and copy number data to deduce stimulation elements in a superimposed pathway network. This network consists of 1,387 pathways.

### RNA sequencing

Cells were treated with dexamethasone (100 nM) or fulvestrant (100 nM) and total RNA was isolated using the RNeasy Mini Kit (Qiagen, Germany) according to the manufacturer's instructions. The quality and quantity of the total RNA were assessed by the 2100 Bioanalyzer using a Nanochip (Agilent, USA). Total RNA samples having an RNA integrity number (RIN) above 8 were subjected to library generation. Strand‐specific libraries were generated with the TruSeq Stranded mRNA sample preparation kit (Illumina, Part # 15031047 Rev. E) and sequenced on a HiSeq2500. RNA‐sequencing data were mapped to exons using Tophat (v.2.1). Read counting, normalization, and differential gene expression were performed using R package DESeq2. Gene‐set enrichment analysis and gene‐list analysis were executed according to the instructions (Subramanian *et al*, 2005).

### ChIP sequencing

Chromatin immunoprecipitations were performed as previously described (Prekovic *et al*, 2021). Nuclear lysates were incubated with 7.5 μl of GR antibody (D6H2L, Cell Signaling Technology) or 5 μg of ER antibody (06‐935, Merck) pre‐bound to 50 μl of protein A beads per sample. Immunoprecipitated DNA was processed for library preparation (0801‐0303, KAPA biosystems kit). Samples were sequenced using an Illumina Hiseq2500 genome analyzer (65 bp reads, single end) and aligned to the Human Reference Genome (hg38). Reads were filtered based on MAPQ quality (samtools v1.8; quality ≥ 20) and duplicate reads were removed (Picard MarkDupes v2.18). Peak calling over input control was performed using MACS3 peak caller. MACS3 was run with the default parameters and consensus peak lists made using macs software. Genome browser snapshots, heatmaps, and density plots were generated using EaSeq (http://easeq.net).

### Rapid immunoprecipitation of endogenous proteins (RIME)

Following hormone deprivation and treatment with dexamethasone (100 nM) for 2 h, RIME experiments were performed as previously described (Prekovic *et al*, 2021). The following antibodies were used: anti‐GR (12041, Cell Signaling Technology), anti‐ERα (06‐935, Merck), and anti‐rabbit IgG (sc‐2027, Santa Cruz Biotechnology). For mass spectrometry, peptide mixtures were prepared and measured as previously described (Prekovic *et al*, 2021), with the following exceptions. Peptide mixtures (10% of total digest) were loaded directly onto the analytical column and analyzed by nanoLC‐MS/MS on an Orbitrap Fusion Tribrid mass spectrometer equipped with a Proxeon nLC1200 system (Thermo Scientific). Solvent A was 0.1% formic acid/water and solvent B was 0.1% formic acid/80% acetonitrile. For GR‐RIME, peptides were eluted from the analytical column at a constant flow of 250 nl/min in a 120 min gradient, containing a 105 min stepped increase from 7 to 34% solvent B, followed by a 15 min wash at 80% solvent B. For ER‐RIME, peptides were eluted from the analytical column at a constant flow of 250 nl/min in a 140 min gradient, containing a 115 min stepped increase from 6 to 32% solvent B, followed by a 25 min wash at 80% solvent B.

Raw data were analyzed by MaxQuant (Cox *et al*, 2014) (version 2.0.1.0) using standard settings for label‐free quantitation (LFQ). MS/MS data were searched against the Swissprot Human database (#501: 20,379 entries, release 2021_01; #522: 20,375 entries, release 2021_04) complemented with a list of common contaminants and concatenated with the reversed version of all sequences. The maximum allowed mass tolerance was 4.5 ppm in the main search and 0.5 Da for fragment ion masses. False discovery rates for peptide and protein identification were set to 1%. Trypsin/P was chosen as cleavage specificity allowing two missed cleavages. Carbamidomethylation was set as a fixed modification, while oxidation and deamidation were used as variable modifications. LFQ intensities were log_2_ transformed in Perseus (Tyanova *et al*, 2016) (version 1.6.15.0), after which proteins were filtered for at least 65% valid values in at least one sample group. Missing values were replaced by imputation based on a normal distribution (width: 0.3 and downshift: 1.8). Differentially expressed proteins were determined using a Student's *t*‐test (minimal threshold: FDR: 5% and S0: 0.1).

### Immunofluorescence and quantification

200,000 MCF‐7 cells and 400,000 EFM‐192A, MDA‐MB‐361, and ZR‐75‐30 cells were seeded on coverslips in 12‐well plates and were designated to treatment or vehicle conditions. Treated cells were maintained in 100 nM dexamethasone for 24 h. Afterward, cells were fixed for 10 min in 2% paraformaldehyde (PFA, 103999, Merck), washed two times in PBS, and were subsequently permeabilized with 0.5% Triton (X100, Sigma Aldrich) diluted in PBS. Following two washing steps with PBS, cells were blocked in 1% BSA/PBS solution, after which they were incubated with primary antibodies against GR (12041, Cell Signaling Technology, 1:50) for 2 h. Alexa Fluor™ 488 goat anti‐rabbit IgG (H+L) cross‐adsorbed secondary antibody (A‐11008, Thermo Fisher Scientific), Alexa Fluor™ 647 goat anti‐rabbit IgG (H+L) cross‐adsorbed secondary antibody (A‐21224, Thermo Fisher Scientific), and ProLong™ Gold antifade reagent with DAPI (P36931, Thermo Fisher Scientific) were used after three washes with PBS to be able to visualize stained cells with laser confocal microscopy (SP5, Leica). Six to twelve images per condition, derived from three independent biological replicates, were analyzed with the Intensity Ratio Nuclei Cytoplasm Tool (RRID:SCR_018573) in ImageJ to determine the ability of GR to translocate to the cell nucleus. Nuclei:cytoplasm ratios of GR localization were quantified by using the averaged nuclei and cytoplasm intensity generated by the ImageJ implemented macro and were normalized to non‐treated conditions.

### Proteomics

Cells were cultured with 100 nM dexamethasone or left untreated for 2, 5, and 7 days. Cells were then collected using cold PBS and after centrifugation pellets were stored at −80°C. Four biological replicates were performed. For protein digestion, frozen cell pellets were lysed in boiling guanidine (GuHCl) lysis buffer as previously described (Jersie‐Christensen *et al*, 2016). Protein concentration was determined with a Pierce Coomassie (Bradford) Protein Assay Kit (Thermo Scientific), according to the manufacturer's instructions. After dilution to 2 M GuHCl, aliquots corresponding to 200 μg of protein were digested twice (overnight and 4 h) with trypsin (Sigma‐Aldrich) at 37°C, enzyme/substrate ratio 1:75. Digestion was quenched by the addition of FA (final concentration 5%), after which the peptides were desalted on a Sep‐Pak C18 cartridge (Waters, Massachusetts, USA). Samples were dried in a vacuum centrifuge and reconstituted in 2% formic acid for MS analysis.

All spectra were acquired on an Orbitrap Exploris 480 Mass Spectrometer (Thermo Fisher Scientific) operated in data‐independent mode (DIA) coupled to an EASY‐nLC 1200 liquid chromatography pump (Thermo Fisher Scientific). Samples were directly loaded onto the analytical column (ReproSil‐Pur 120 C18‐AQ, 2.4 μm, 75 μm × 500 mm, packed in‐house). Solvent A was 0.1% formic acid/water and solvent B was 0.1% formic acid/80% acetonitrile. For dexamethasone time course experiment in MCF‐7 cells, samples were eluted from the analytical column at a constant flow of 250 nl/min in a 90 min gradient, containing a 78 min linear increase from 6 to 30% solvent B, followed by a 12 min wash at 90% solvent B. For ZBTB16‐OE experiments, samples were eluted from the analytical column at a constant flow of 250 nl/min in a 70 min gradient, containing a 58 min linear increase from 7 to 30% solvent B, followed by a 12 min wash at 90% solvent B.

Raw data were analyzed by DIA‐NN (Demichev *et al*, 2020) (version 1.8) without a spectral library and with “Deep learning” option enabled. The Swissprot human database (#562: 20,375 entries, release 2022_02; #615: 20,398 entries, release 2022_08) was added for the library‐free search. The quantification strategy was set to Robust LC (high accuracy) and MBR option was enabled. The other settings were kept at the default values. The protein groups report from DIA‐NN was used for downstream analysis in Perseus (#562: version: 1.6.15.0; #615: version: 2.0.7.0).

Values were log_2_ transformed, after which proteins were filtered for at least 75% valid values in at least one sample group. Missing values were replaced by imputation based on a normal distribution using a width of 0.3 and a minimal downshift of 2. Differentially expressed proteins were determined using a Student's *t*‐test with multiple‐testing corrections.

### Viability assay

Cells were seeded into 384‐well plates and treated with compounds using the HP D300 Digital Dispenser. At least three technical replicates were performed for each drug, and phenylarsine oxide (PAO) and dimethylsulfoxide (DMSO) were used as controls. Following 5‐day treatment, a CellTiter‐Glo (CTG; Promega, Madison, WI, USA) assay was performed. Following the manufacturer's instructions, cell viability was determined based on luminescent output detected using a Tecan microplate reader.

### Western blot

Cells were lysed using 2× Laemmli buffer (120 mM Tris, 20% glycerol, 4% SDS) supplemented with protease inhibitor (1:100) and phenylmethylsulfonyl fluoride (PMSF, 1:200). Lysates were sonicated for 10 cycles with 1 s intervals and a 20% amplitude. Equal amounts of protein per lysate were run for 1 h at 100 volts on an 8% acrylamide gel (MilliQ, 40% acrylamide, 1.5 M Tris pH 6.8, 10% SDS, and 10% APS, TEMED) in SDS–PAGE 1× running buffer (25 mM Tris, 0.25 M glycine, and 0.1% SDS). Proteins were transferred on ice at 100 volts for 90 min or at 0.9 mA overnight at 4°C on a nitrocellulose membrane in cold 1× transfer buffer (24 mM Tris and 192 mM glycine). Membranes were blocked in 3% BSA (A8022, Sigma/Merck) in 1× PBS‐Tween (137 mM NaCl, 10 mM Na2HPO4, 1.5 mM KH2PO4, 2.6 mM KCl, and 0.1% Tween‐20) for 1 h and incubated with primary antibodies against V5 (R960‐25, ThermoFisher) diluted in 3% BSA/PBS‐T for 2 h. After three washing steps in PBS‐T, membranes were incubated with secondary antibodies donkey‐α‐mouse 680 RD (926‐68073, LI‐COR Biosciences, 1:10,000), diluted in 3% BSA/PBS‐T for 1 h. Membranes were scanned and analyzed using an Odyssey® CLx Imaging System (LI‐COR Biosciences) and ImageStudio™ Lite v.5.2.5 software (LI‐COR Biosciences).

### Xenograft study

MCF‐7 cells were trypsinized and resuspended in PBS at a density of 25,000 cells/20 μl. Female NOD‐scid‐γ (NSG; The Jackson Laboratory) mice (±8 weeks old) were anesthetized before injection of cells into four mammary glands. Once the tumor size reached 100 mm^3^, the mice were randomized into groups, ensuring blinding to the treatment conditions. Blinding was maintained throughout the experiment and data analysis period. Inclusion criteria for the study comprised of the mice successfully developing tumors of the specified size, while exclusion criteria included any mice showing signs of severe distress or other unrelated health issues.

Treated mice received 4 mg/kg dexamethasone (D2915‐100MG, Sigma‐Aldrich; dissolved in water) or vehicle by I.P. injections three times per week. Additionally, β‐estradiol (4 μg/ml) was given via drinking water (changed once per week); treatment started 7 days prior to intraductal injections and was maintained until the end of the study or the 7‐month end time point. Tumor volume was monitored by caliper measurements every 2–3 days.

Mice were housed under standard temperature and humidity conditions in individually ventilated cages, with food and water provided *ad libitum*. The NKI Animal Experiments Committee approved the *in vivo* experiments under project number 10537.

### Single‐cell RNA‐sequencing analysis

Processed single‐cell RNA‐sequencing data from 26 breast cancer patients (Wu *et al*, 2021) was downloaded from the Broad Institute Single Cell portal, at https://singlecell. broadinstitute.org/single_cell/study/SCP1039. AUCell v1.4.1 (Aibar *et al*, 2017) was used to calculate the pathway enrichment for each individual cell for both the derived GR activity signature and a curated set of breast cancer‐related gene signatures (Wu *et al*, 2021). From this single‐cell dataset, 12,147 neoplastic cells, with either a Luminal A (LumA_SC) or Luminal B (LumB_SC) scSubtype annotation (Wu *et al*, 2021), were extracted.

### Organoid experiments

Organoids were generated from tumors collected and processed with IRB approval from the RCSI University of Medicine and Health Sciences. HCI05 and HCI‐011 models were a kind gift from the Alana Welm lab. The protocol for establishing the organoid lines utilized was previously described (Cosgrove *et al*, 2022; Guillen *et al*, 2022). Briefly, established organoids were dissociated into single cells and seeded in organoid media with 5% Cultrex® Reduced Growth Factor Basement Membrane Matrix (Trevigen, 3533‐001‐02). Twenty‐four hours after seeding, the organoids were treated with either vehicle or indicated compounds (all sourced from MedChemExpress) (*n* = 6). Cell viability was measured 7 days post‐treatment using the CellTiter‐Glo® 3D Cell Viability assay (Promega).

### Statistical analysis

All statistical analysis was performed using R. Normality was tested using D'Agostino and Shapiro–Wilk test. Technique‐specific statistical tests are described within their corresponding method subsection.

## Author contributions

**Stefan Prekovic:** Conceptualization; resources; data curation; formal analysis; supervision; funding acquisition; validation; investigation; visualization; methodology; writing – original draft; project administration; writing – review and editing. **Theofilos Chalkiadakis:** Data curation; software; formal analysis; validation; investigation; visualization; methodology; writing – review and editing. **Merel Roest:** Formal analysis; validation; investigation; visualization; methodology; writing – review and editing. **Daniel Roden:** Data curation; formal analysis; methodology; writing – review and editing. **Catrin Lutz:** Formal analysis; investigation; methodology; writing – review and editing. **Karianne Schuurman:** Investigation; methodology. **Mark Opdam:** Resources; data curation. **Liesbeth Hoekman:** Investigation; methodology. **Nina Abbott:** Investigation; methodology. **Tanja Tesselaar:** Investigation; methodology. **Maliha Wajahat:** Investigation; methodology. **Amy R Dwyer:** Investigation; methodology. **Isabel Mayayo‐Peralta:** Investigation; methodology. **Gabriela Gomez:** Investigation; methodology. **Maarten Altelaar:** Investigation; methodology. **Roderick Beijersbergen:** Investigation; methodology. **Balázs Győrffy:** Data curation; investigation. **Leonie Young:** Resources; investigation; methodology. **Sabine Linn:** Resources; investigation. **Jos Jonkers:** Investigation; methodology. **Wayne Tilley:** Investigation; methodology. **Theresa Hickey:** Investigation; methodology. **Damir Vareslija:** Resources; formal analysis; investigation; methodology. **Alexander Swarbrick:** Investigation; methodology. **Wilbert Zwart:** Resources; supervision; funding acquisition; investigation; project administration; writing – review and editing.

## Disclosure and competing interests statement

SL receives research funding to her institution from Agendia, AstraZeneca, Eurocept pharmaceuticals, Genentech, GSK (formerly Tesaro), Immunomedics, Merck, Novartis, and Roche; has acted as a consultant (not compensated) for Cergentis and Philips Health; has acted as a consultant (paid to her institution) for AstraZeneca and IBM; and has received educational funding to her institution from Bayer and Daiichi‐Sankyo. In addition, SL has a patent UN23A01/P‐EP pending. Other authors declare no competing interests.

## Supporting information



Expanded View Figures PDF

Table EV1

Table EV2

PDF+

Source Data for Figure 3

Source Data for Figure 4

## Data Availability

All genomic and mass spectrometry data generated in this study have been deposited in the Gene Expression Omnibus (GEO) and Proteomics Identification (PRIDE) databases, under accession numbers GSE222799 (http://www.ncbi.nlm.nih.gov/geo/query/acc.cgi?acc=GSE222799) and PXD039209 (http://www.ebi.ac.uk/pride/archive/projects/PXD039209), respectively.
