# Supplementary material for: Luminal breast cancer identity is determined by loss of glucocorticoid receptor activity
Source: EMBO Mol Med. 2023 Oct 30;15(12):e17737. doi: 10.15252/emmm.202317737 (PMC10701603; doi:10.15252/emmm.202317737)
Supplement: Supplementary file 1 — Expanded View Figures PDF [file EMMM-15-e17737-s002.pdf]

Expanded View Figures

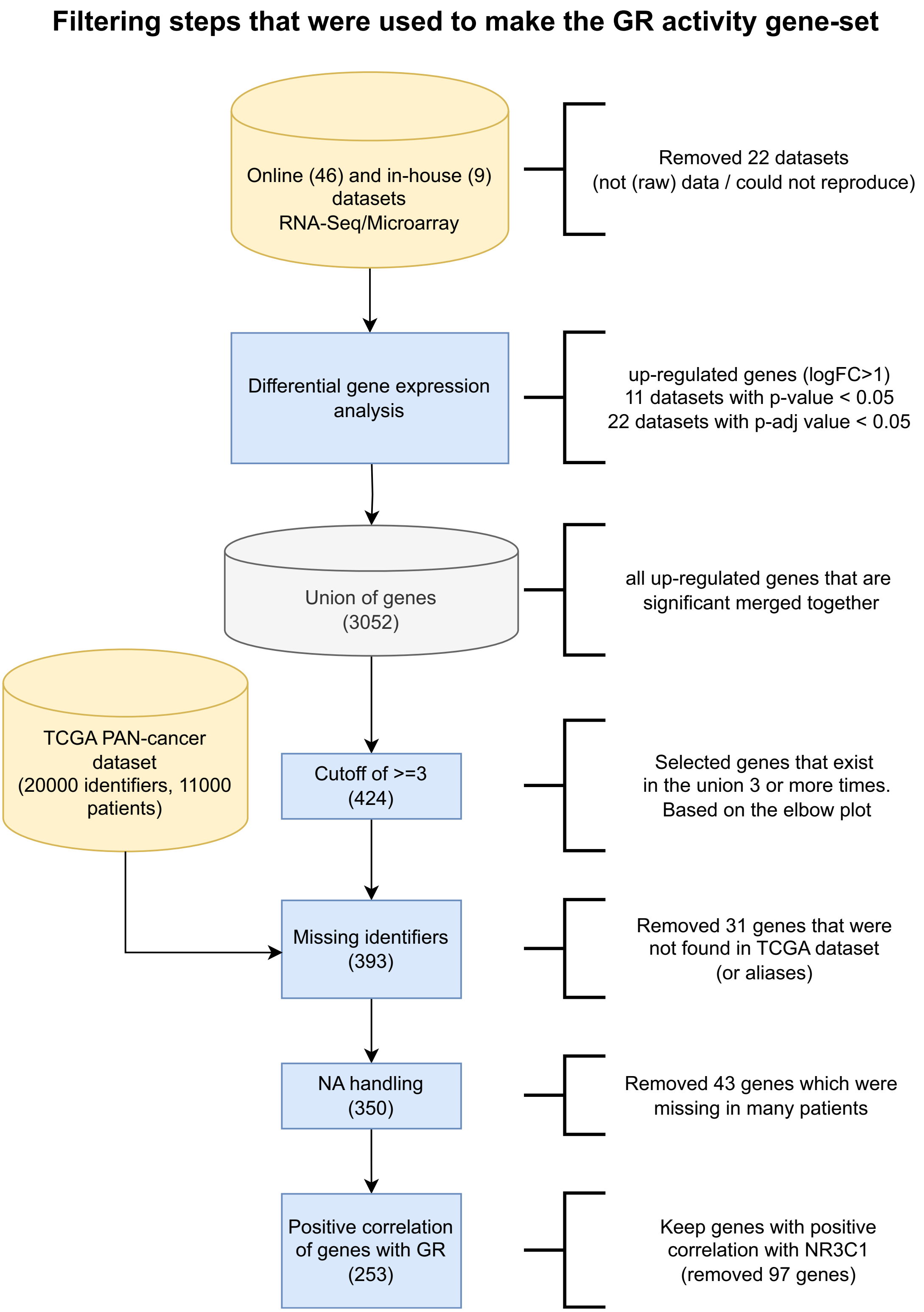

Figure EV1.

© 2023 Netherlands Cancer Institute

Figure EV1. The scheme representing the process and filtering steps taken to make the GR activity signature.

Figure EV2. Optimization of the GR activity signature.

A Elbow plot depicting overlap between models. This analysis aided refinement of the signature to a consensus list of number of genes shared among at least three independent models, yielding a total list of 424 genes.
B Expression correlation analyses of NR3C1 mRNA levels with expression levels of genes represented in the signature in cancer samples. Only positively correlating genes were included for further analyses, yielding a final set of 253 genes.
C Enrichment of GR binding in vicinity of GRa (right, full line) or random (left, dashed line; n = 1,000 iterations; error bars represent mean ± SD) genes in ChIP-sequencing experiments from various cell lines (full lines, A549, BEAS-2B, HeLa, LNCaP, MCF-7, THP1, U2OS, and ZR-75-1).
D Overlap of GR activity signature, with ER (gene-set reference: M5906 and M5907) and AR (gene-set reference: M5908) gene signatures.
E Correlation of NR3C1 mRNA levels with GR activity across samples represented in the TCGA dataset. Color indicates the number of samples in each bin.
F Correlation of GR mRNA levels with random gene set of equivalent size to GR activity signature among samples represented in the TCGA dataset. Color indicates the number of samples in each bin.
G Histogram depicting correlation of random gene set of equivalent size to GR activity signature with NR3C1 mRNA levels in TCGA cancers; black line depicts the correlation of GR activity with NR3C1 mRNA levels in the same cohort.
H Overrepresentation analysis of various gene sets within the GR activity signature. Each point represents a different pathway.
I Pearson correlation analysis of single-sample gene-set enrichment analysis (ssGSEA) scores for each of the TCGA samples (n = 9,829) and GR activity. Representation of the different tumor types is depicted below.
J Normalized (phospho)protein expression in human breast cancer samples of the TCGA (n = 747) grouped based on GR activity. The central mark indicates the median, and the bottom and top edges of the box indicate the 25th and 75th percentiles, respectively. The maximum whisker lengths are specified as 1.5 times the interquartile range.

© 2023 Netherlands Cancer Institute

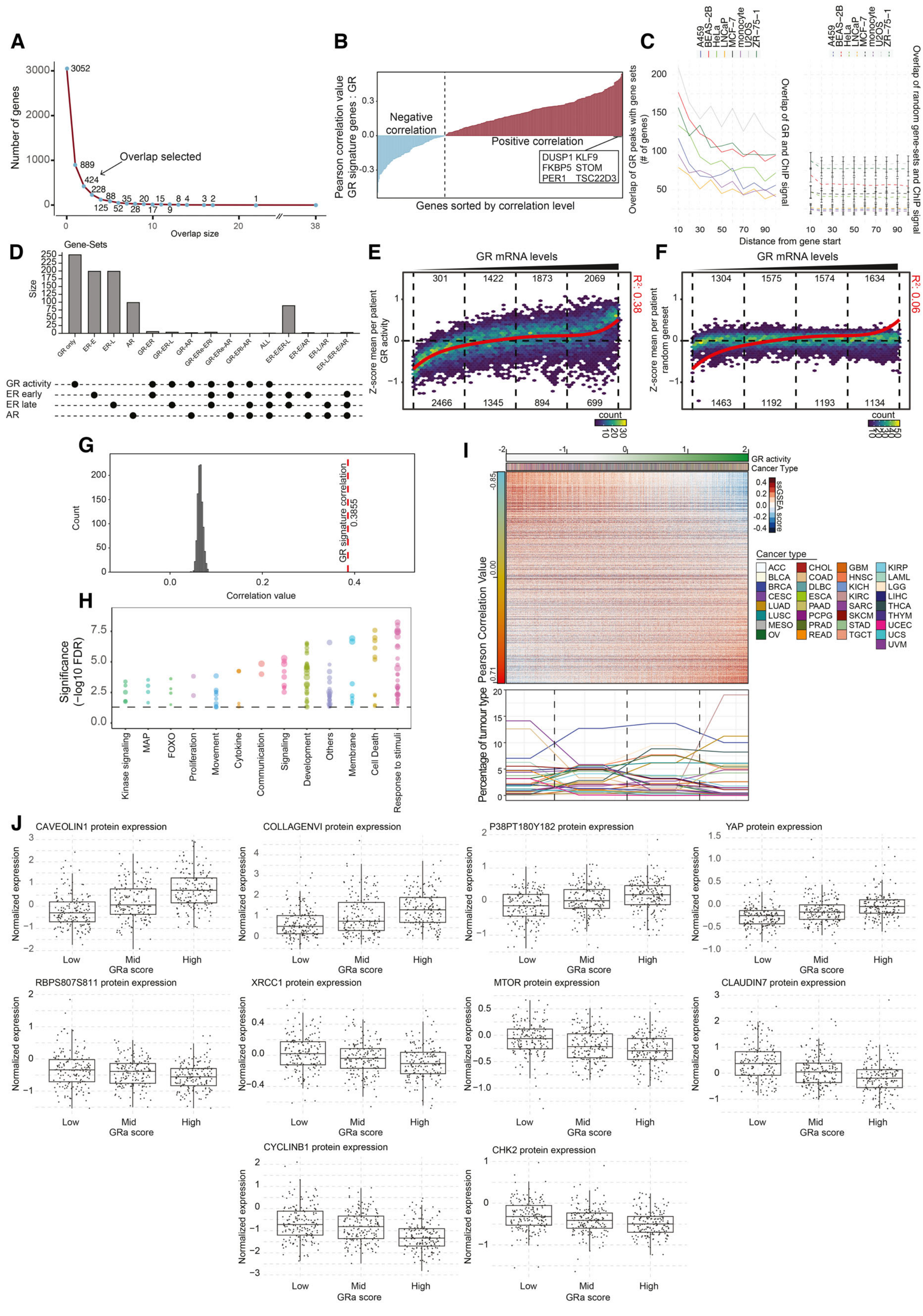

Figure EV2.

© 2023 Netherlands Cancer Institute

Figure EV3. GR activity in breast cancer is linked to luminal subtype status and survival probabilities.

A Boxplots depicting GR activity signature in Luminal A and B cancers of the METABRIC cohort (n = 1,134 luminal samples). The box begins in the first quartile (25%) and ends in the third (75%), while the line represents the median value. The lines represent segments to furthest data without accounting for outliers. P-values were determined by the Wilcoxon t-test.

B Boxplots depicting GR activity signature in Luminal A and B cancers of the MATADOR trial (n = 415 luminal samples). P-values were determined by the Wilcoxon t-test. The box begins in the first quartile (25%) and ends in the third (75%), while the line represents the median value. The lines represent segments to furthest data without accounting for outliers.

C The UMAP analysis of time-course experiments performed with T47D cell line utilizing the GRa genes. Each cell is colored according to the treatment time points—0, 1, 2, 4, 8, and 18 h.

D Boxplot depicting the value of GR activity per cell per time point of the time course (400 cells). The central mark indicates the median, and the bottom and top edges of the box indicate the 25th and 75th percentiles, respectively. The lines represent segments to furthest data without accounting for outliers.

E Average gene expression of genes corresponding to clusters identified by k-means clustering of PAM50 genes in luminal breast cancer patient samples. The box begins in the first quartile (25%) and ends in the third (75%), while the line represents the median value. The lines represent segments to furthest data without accounting for outliers.

F Overall survival probabilities of breast cancer patients (n = 1,310) grouped by transcriptomics-based 8-gene classifier (Luminal A high expression = purple, Luminal A low expression = gray, and Luminal B = blue). Overall survival probabilities in months are plotted for each group, and censored patients are shown as vertical tick marks.

G Prognostic power as determined by SigCheck of the 8-gene classifier (red dotted line) with 1,000 random gene sets of the same size (P-value < 0.05 is indicated by the blue dotted line) for overall survival parameter in KMplotter breast cancer cohort (n = 1,310).

H Overall survival probabilities of breast cancer patients (TCGA cohort n = 280) grouped by transcriptomics-based 8-gene classifier (Luminal A high expression = purple, Luminal A low expression = gray, and Luminal B = blue). Overall survival probabilities in months are plotted for each group, and censored patients are shown as vertical tick marks. First 300 months were included in the analysis.

I Overall survival probabilities of breast cancer patients (KMplotter cohort n = 3,772; TCGA cohort n = 280) grouped by GR activity signature (Luminal A high expression = purple, Luminal A low expression = gray, and Luminal B = blue). Overall survival probabilities in months are plotted for each group, and censored patients are shown as vertical tick marks. First 300 months were included in the analysis.

© 2023 Netherlands Cancer Institute

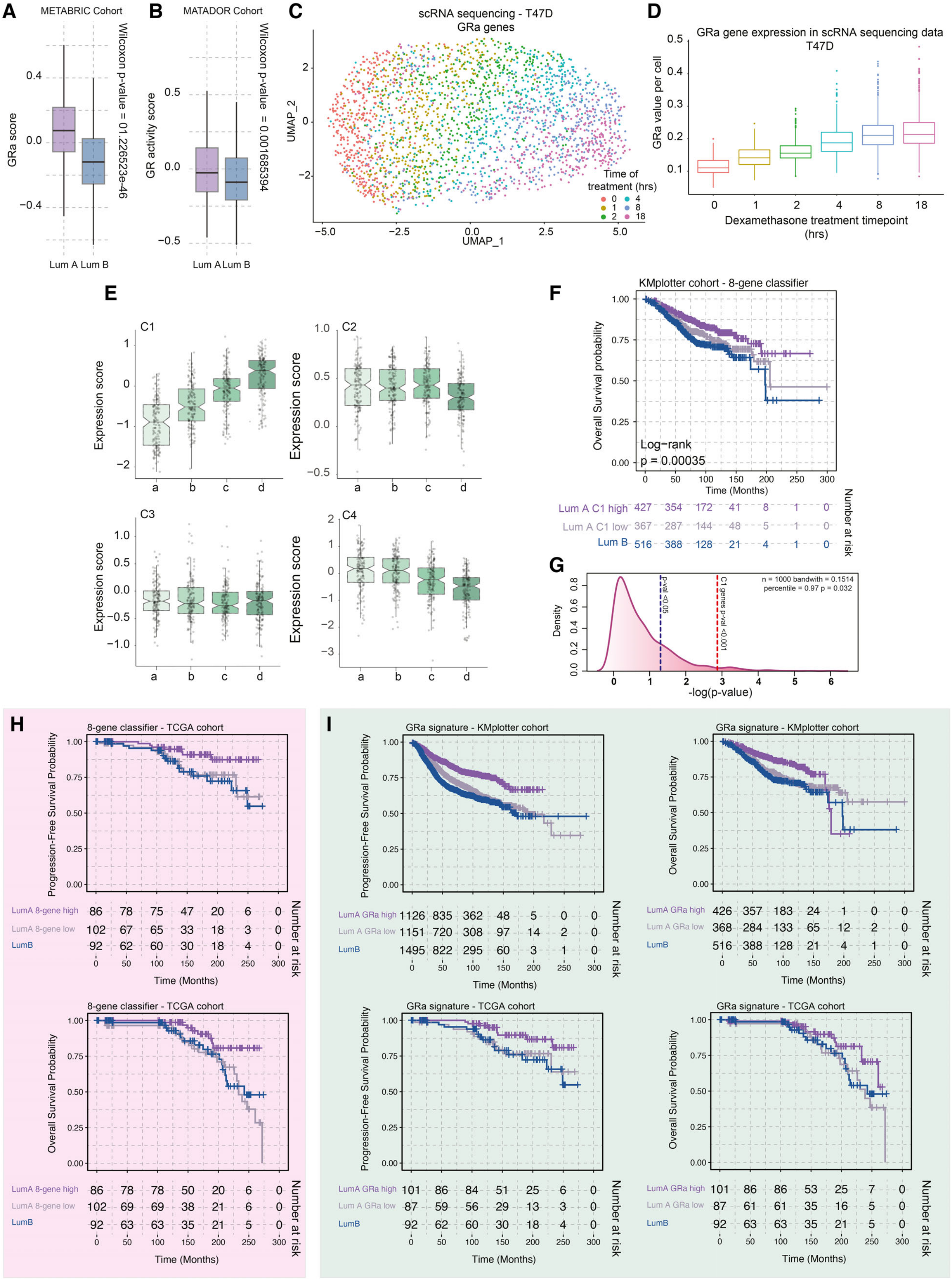

Figure EV3.

© 2023 Netherlands Cancer Institute

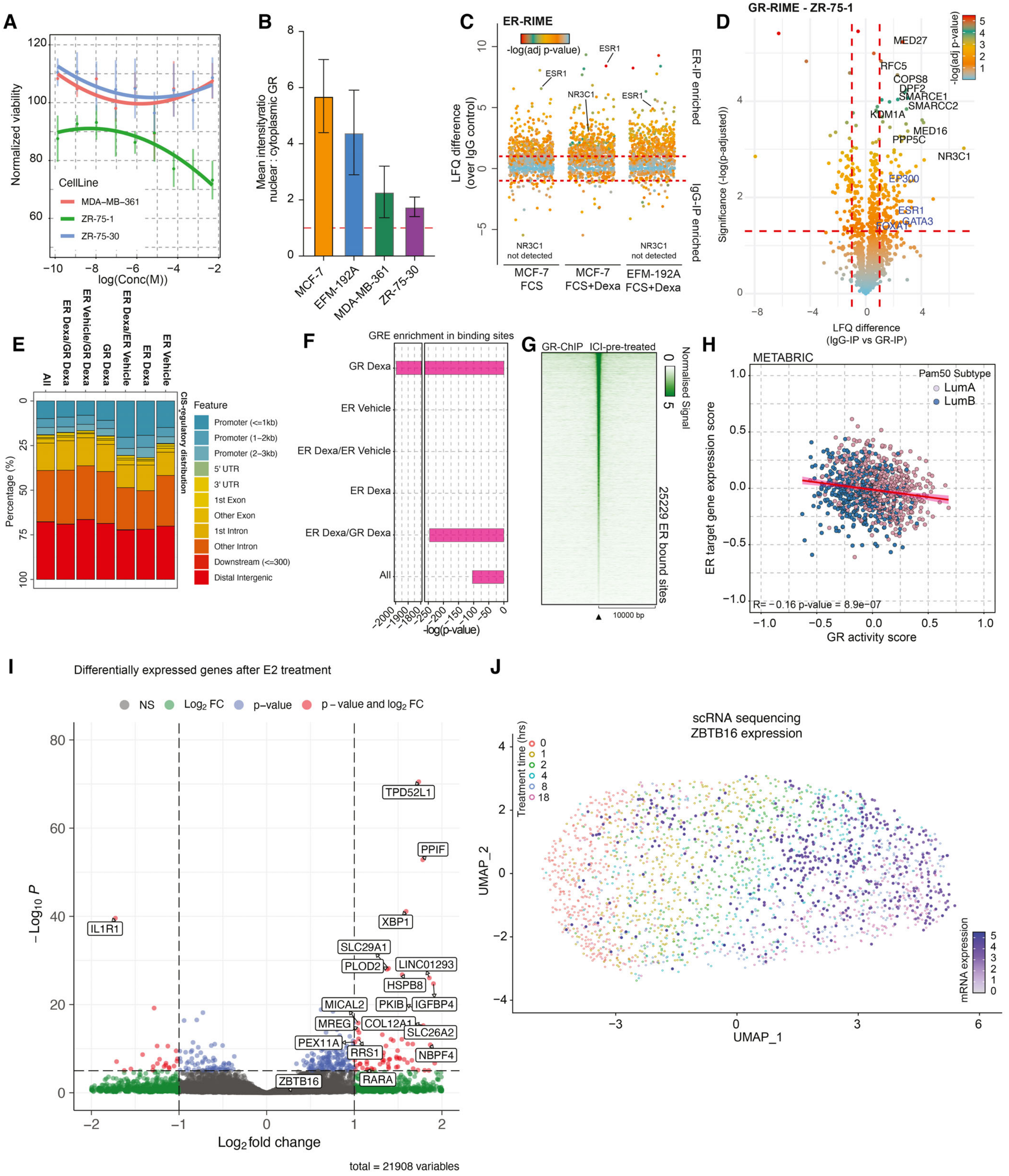

A
Normalized viability
log(Conc(M))
CellLine
MDA–MB–361
ZR–75–1
ZR–75–30
B
Mean intensityratio nuclear : cytoplasmic GR
MCF-7
EFM-192A
MDA-MB-361
ZR-75-30
C
ER-RIME
-log(adj p-value)
LFQ difference (over IgG control)
ER-IP enriched
IgG-IP enriched
NR3C1 not detected
MCF-7 FCS
MCF-7 FCS+Dexa
EFM-192A FCS+Dexa
D
GR-RIME - ZR-75-1
Significance (-log(p-adjusted))
LFQ difference (IgG-IP vs GR-IP)
-log(adj p-value)
MED27
RFC5
COPS8
DPF2
SMARCE1
SMARCC2
KDM1A
MED16
PPP5C
NR3C1
EP300
ESR1
GATA3
FOXA1
E
All
ER Dexa/GR Dexa
ER Vehicle/GR Dexa
GR Dexa
ER Dexa/ER Vehicle
ER Dexa
ER Vehicle
Percentage (%)
CIS-regulatory distribution
Feature
Promoter (<=1kb)
Promoter (1–2kb)
Promoter (2–3kb)
5' UTR
3' UTR
1st Exon
Other Exon
1st Intron
Other Intron
Downstream (<=300)
Distal Intergenic
F
GRE enrichment in binding sites
GR Dexa
ER Vehicle
ER Dexa/ER Vehicle
ER Dexa
ER Dexa/GR Dexa
All
-log(p-value)
G
GR-ChIP ICI-pre-treated
Normalised Signal
25229 ER bound sites
10000 bp
H
METABRIC
Pam50 Subtype
LumA
LumB
ER target gene expression score
GR activity score
R= −0.16 p-value = 8.9e−07
I
Differentially expressed genes after E2 treatment
NS
Log2 FC
p–value
p – value and log2 FC
– Log10 P
Log2 fold change
total = 21908 variables
TPD52L1
PPIF
IL1R1
XBP1
SLC29A1
PLOD2
LINC01293
HSPB8
MICAL2
PKIB
IGFBP4
MREG
COL12A1
SLC26A2
PEX11A
RRS1
NBPF4
ZBTB16
RARA
J
scRNA sequencing
ZBTB16 expression
Treatment time (hrs)
UMAP_2
UMAP_1
mRNA expression

Figure EV4.

Figure EV4. Molecular aspect of GR signaling in luminal breast cancer.

A Normalized cancer cell viability (lines depict polynomial fit) in response to GR agonist (Dexa) treatment. $n = 4$. Mean values ± SEM depicted.
B Bar plot depicting nuclear-to-cytoplasmic ratio of GR in various cell lines. $n = 3$. Mean values ± SEM depicted.
C Jitter plot depicting differentially enriched interactors in ER-RIME experiments between Veh-treated MCF-7 and Dexa-treated MCF-7 and EFM-192A cell lines. $n = 4$. *P*-values were determined by two-sided *t*-test.
D Volcano plot depicting enriched (in comparison to IgG-IP control) interactors in GR-RIME experiments in ZR-75-1 cell line. $n = 3$. *P*-values were determined by two-sided *t*-test.
E Genomic distribution analyses for all the sites detected in ChIP-sequencing experiments. $n = 3$.
F HOMER motif analysis of *P*-value for GREs across different categories of sites.
G Heatmap of ChIP-sequencing signal for GR around peak midpoint for all sites detected across the genome after 24 h pre-treatment with ICI and subsequent 2 h treatment with Dexa. $n = 3$.
H Scatterplot depicting the absence of correlation of GR activity with ER target gene expression in luminal breast cancers.
I Volcano plot depicting transcriptomic differences between DCC-treated and DCC+E2-treated MCF-7 ($n = 4$). Adjusted *P*-values were determined by DESeq2 (Wald test *P*-values corrected for multiple testing using Benjamini and Hochberg method).
J The UMAP analysis of time-course experiments performed with T47D cell line utilizing the GRa genes. Each cell is colored according to the treatment time points—0, 1, 2, 4, 8, and 18 h, and the values of *ZBTB16* gene expression are projected on top (white-to-blue gradient).

Data information: All experiments were performed in biological replicate and the number (*n*) of replicates indicated.

Figure EV5. *ZBTB16* action in ER-positive breast cancer.

A Boxplot depicting *ZBTB16* mRNA expression in normal breast tissues, Luminal A, as well as Luminal B cancer samples (TCGA cohort $n = 702$). The box begins in the first quartile (25%) and ends in the third (75%), while the line represents the median value. The lines represent segments to furthest data without accounting for outliers.
B Overall survival probabilities of breast cancer patients (KMplotter meta dataset; $n = 1,310$) grouped by *ZBTB16* mRNA expression (Luminal A high expression = purple, Luminal A low expression = gray, and Luminal B = blue). Overall survival probabilities in months are plotted for each group, and censored patients are shown as vertical tick marks.
C Progression-free and overall survival probabilities of breast cancer patients (METABRIC dataset) grouped by *ZBTB16* mRNA expression (Luminal A high expression = purple, Luminal A low expression = gray, and Luminal B = blue). Progression-free and overall survival probabilities in months are plotted for each group, censored patients are shown as vertical tick marks.
D Western blot showing expression of V-5-tagged GFP and ZBTB16. $n = 3$.
E Normalized cancer cell viability for MCF-7 GFP (green) and ZBTB16 (orange) overexpression models. $n = 4$.
F Representative crystal violet assay image for MCF-7 GFP and ZBTB16 overexpression models. $n = 3$.
G Upper panel, volcano plot depicting differences in protein expression between GFP-OE and ZBTB16-OE models in MCF-7 cells. Significance is depicted as a color gradient; lower panel, intensity (LFQ) values per category (decrease in expression, same levels, and increase in expression upon ZBTB16 overexpression). $n = 4$. The box begins in the first quartile (25%) and ends in the third (75%), while the line represents the median value. The lines represent segments to furthest data without accounting for outliers.
H Volcano plot depicting differences in protein expression between GFP-OE and ZBTB16-OE models in EFM-192A cells. $n = 4$.
I Reactome cell cycle (gene-set reference: R-HSA-1640170) GSEA enrichment profiles based on whole-proteome data comparison between GFP-OE and ZBTB16-PE in MCF-7 cell line.
J Hallmark E2F targets (gene-set reference: M5925) GSEA enrichment profiles based on whole-proteome data comparison between GFP-OE and ZBTB16-PE in EFM-192A cell line.
K Volcano plot depicting differentially enriched (over IgG control) interactors in ZBTB16-RIME experiments in MCF-7 cells. $n = 4$. *P*-values were determined by two-sided *t*-test.
L GSEA enrichment profiles for "Reactome Signaling by nuclear receptors" gene sets based on IgG versus ZBTB-16 RIME comparison ($n = 4$).

Data information: All experiments were performed in biological replicates and the number (*n*) of replicates indicated.

© 2023 Netherlands Cancer Institute

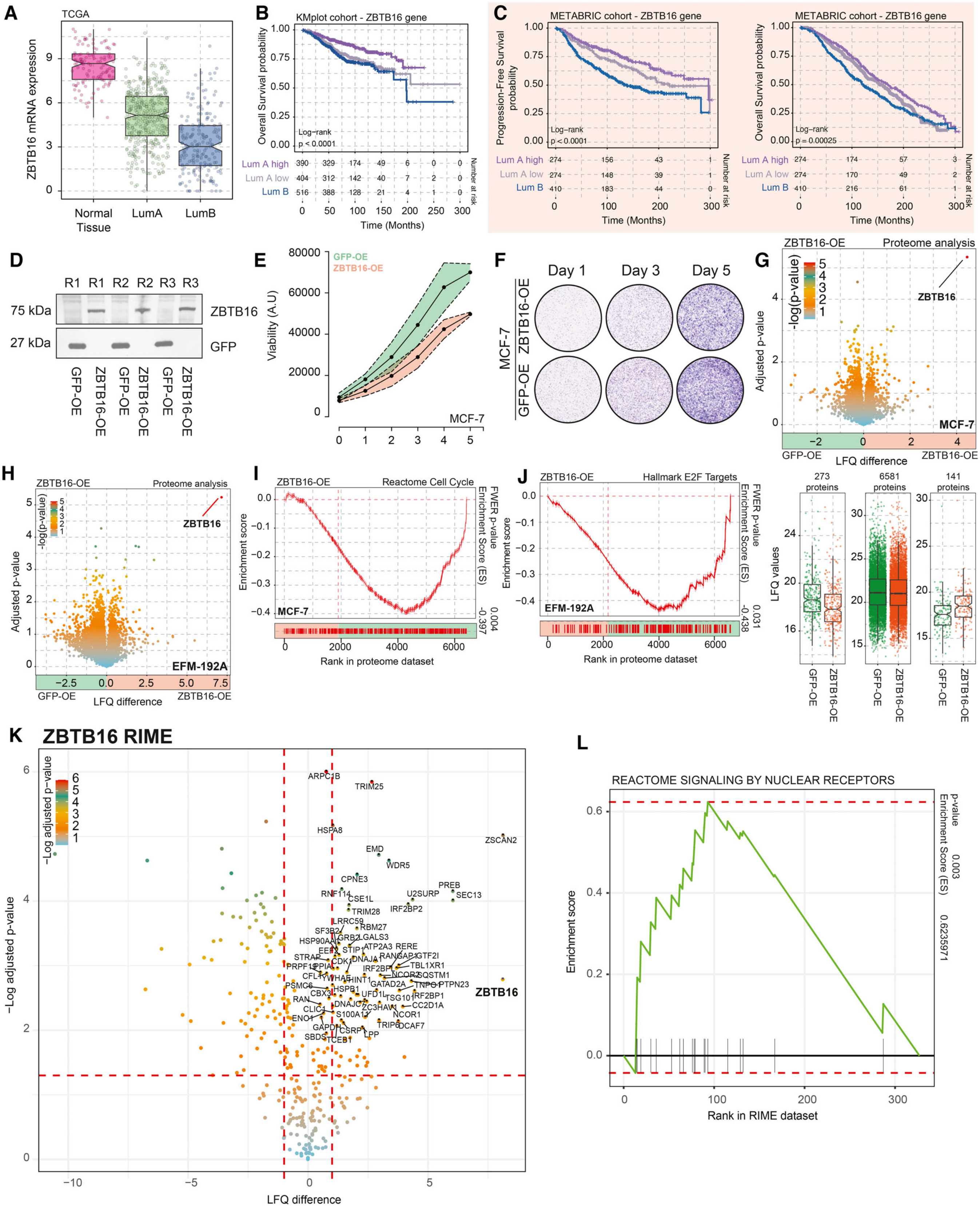
A
TCGA
ZBTB16 mRNA expression
Normal Tissue
LumA
LumB
B
KMplot cohort - ZBTB16 gene
Overall Survival probability
Log−rank
p < 0.0001
Lum A high
Lum A low
Lum B
Number at risk
Time (Months)
C
METABRIC cohort - ZBTB16 gene
Progression-Free Survival probability
Log−rank
p < 0.0001
Overall Survival probability
p = 0.00025
Time (Months)
D
R1 R1 R2 R2 R3 R3
75 kDa
27 kDa
ZBTB16
GFP
GFP-OE
ZBTB16-OE
E
GFP-OE
ZBTB16-OE
Viability (A.U)
MCF-7
F
Day 1
Day 3
Day 5
MCF-7
ZBTB16-OE
GFP-OE
G
ZBTB16-OE
Proteome analysis
-log(p-value)
ZBTB16
Adjusted p-value
MCF-7
LFQ difference
GFP-OE
ZBTB16-OE
H
ZBTB16-OE
Proteome analysis
ZBTB16
Adjusted p-value
EFM-192A
LFQ difference
GFP-OE
ZBTB16-OE
I
ZBTB16-OE
Reactome Cell Cycle
Enrichment score
FWER p-value
Enrichment Score (ES)
MCF-7
0.004
-0.397
Rank in proteome dataset
J
ZBTB16-OE
Hallmark E2F Targets
Enrichment score
FWER p-value
Enrichment Score (ES)
EFM-192A
0.031
-0.438
Rank in proteome dataset
273 proteins
6581 proteins
141 proteins
LFQ values
GFP-OE
ZBTB16-OE
K
ZBTB16 RIME
−Log adjusted p−value
ZBTB16
ARPC1B
TRIM25
HSPA8
ZSCAN2
EMD
WDR5
CPNE3
PREB
RNF114
U2SURP
SEC13
CSE1L
IRF2BP2
TRIM28
LRRC59
RBM27
GALS3
ATP2A3
RERE
STIP1
RANGAP1
GTF2I
TBL1XR1
IRF2BP1
NCOR2
SQSTM1
GATAD2A
TNPO1
PTPN23
TSG101
CC2D1A
NCOR1
TRIP6
DCAF7
LPP
CSRP1
TCEB1
GAPDH
SBDS
ENO1
CLIC1
RAN
S100A11
CBX3
PSMC6
HSPB1
HINT1
CFL1
PPIA
PRPF19
STRAP
LFQ difference
L
REACTOME SIGNALING BY NUCLEAR RECEPTORS
Enrichment score
p-value
Enrichment Score (ES)
0.003
0.6235971
Rank in RIME dataset

Figure EV5.

Figure EV6. Inhibition of epigenetic pathways in additional models of breast cancer.

A Comparison of Dexa-induced proteomic changes in proteomics experiments across different time points (2- vs. 5- vs. 7-day treatment). $n = 4$. Adjusted *P*-values (*P*adj) were determined by *t*-test (*P*-values corrected for multiple testing using Benjamini and Hochberg method). Pearson correlation value is reported.
B Snake plot depicting the gene knockout fitness effects (Chronos) for top genes potentially regulated by vehicle unique H3K27Ac sites.
C Western blot showing expression of GR and ER in all the breast cancer cell line models used in the manuscript, with actin as a loading control ($n = 2$).
D Normalized cancer cell viability in response to BRD inhibitor (Molibresib) treatment. $n = 4$.
E Normalized cancer cell viability in response to BRD inhibitor (Birabresib) treatment. $n = 4$.
F Normalized cancer cell viability in response to HDAC inhibitor (Mocetinostat) treatment. $n = 4$.
G Normalized cancer cell viability in response to HDAC inhibitor (Vorinostat) treatment. $n = 4$.
H Bar chart showing response to various inhibitors for each of the organoid lines alongside key tumor characteristics. Drug concentrations (dexamethasone 100 nM, ribociclib 1,000 nM, alobresib 100 nM, birabresib 500 nM, vorinostat 500 nM, panobinostat 10 nM, and molibresib 500 nM). Error bars represent mean ± SEM. $n = 6$.

Data information: All experiments were performed in biological replicates and the number (*n*) of replicates indicated.

© 2023 Netherlands Cancer Institute

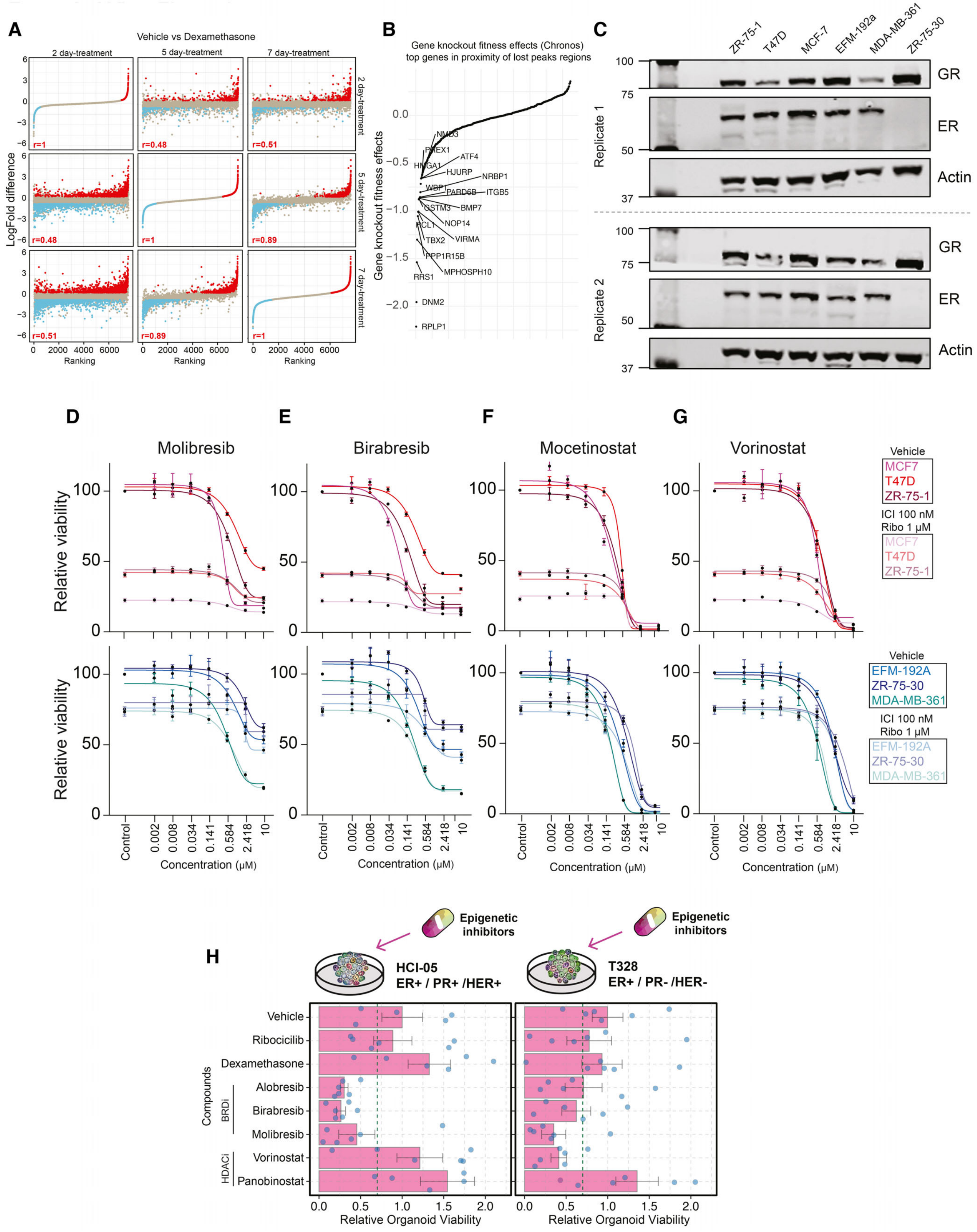
A
Vehicle vs Dexamethasone
2 day-treatment
5 day-treatment
7 day-treatment
LogFold difference
Ranking
B
Gene knockout fitness effects (Chronos)
top genes in proximity of lost peaks regions
Gene knockout fitness effects
C
ZR-75-1
T47D
MCF-7
EFM-192a
MDA-MB-361
ZR-75-30
GR
ER
Actin
Replicate 1
Replicate 2
D
Molibresib
E
Birabresib
F
Mocetinostat
G
Vorinostat
Relative viability
Concentration (µM)
Vehicle
MCF7
T47D
ZR-75-1
ICI 100 nM
Ribo 1 µM
EFM-192A
ZR-75-30
MDA-MB-361
H
Epigenetic inhibitors
HCI-05
ER+ / PR+ /HER+
T328
ER+ / PR- /HER-
Vehicle
Ribocicilib
Dexamethasone
Alobresib
Birabresib
Molibresib
Vorinostat
Panobinostat
Compounds
BRDi
HDACi
Relative Organoid Viability

Figure EV6.
