# Supplementary material for: Luminal breast cancer identity is determined by loss of glucocorticoid receptor activity
Source: EMBO Mol Med. 2023 Oct 30;15(12):e17737. doi: 10.15252/emmm.202317737 (PMC10701603; doi:10.15252/emmm.202317737)
Supplement: Supplementary file 7 [file EMMM-15-e17737-s007.pdf]

EMBO Molecular Medicine

Luminal breast cancer identity is determined by loss of glucocorticoid receptor activity

Stefan Prekovic, Theofilos Chaliadakis, Merel Roest, Daniel Roden, Catrin Lutz, Karianne Schuurman, mark opdam, liesbeth hoekman, Nina Abbott, Tanja Tesselaar, Maliha Wajahat, Amy Dwyer, Isabel Mayayo-Peralta, Gabriela Gomez, Maarten Altelaar, Roderick Beijersbergen, Balázs Győrffy, Leonie Young, Sabine Linn, Jos Jonkers, Wayne Tilley, Theresa Hickey, Damir Vareslija, Alexander Swarbrick, and Wilbert Zwart
DOI: 10.15252/emmm.202317737

Corresponding authors: Wilbert Zwart (w.zwart@nki.nl) , Stefan Prekovic (s.prekovic@umcutrecht.nl)

Review Timeline:

Submission Date:	21st Mar 23
Editorial Decision:	4th May 23
Revision Received:	14th Aug 23
Editorial Decision:	13th Sep 23
Revision Received:	27th Sep 23
Accepted:	4th Oct 23

Editor: Lise Roth

Transaction Report:

(Note: With the exception of the correction of typographical or spelling errors that could be a source of ambiguity, letters and reports are not edited. Depending on transfer agreements, referee reports obtained elsewhere may or may not be included in this compilation. Referee reports are anonymous unless the Referee chooses to sign their reports.)

4th May 2023

Dear Prof. Zwart,

Thank you for the submission of your manuscript to EMBO Molecular Medicine, and please accept my apologies for the delay in getting back to you, as one referee asked to be withdrawn due to personal issues and had to be replaced. We have now received the reports from the three referees who eventually reviewed your manuscript. As you will see below, the reviewers raise substantial concerns on your work, which unfortunately preclude its publication in EMBO Molecular Medicine in its current form.

The reviewers find that the question addressed by the study is of potential interest, however they remain unconvinced that some of the major conclusions are sufficiently supported by the data. In particular, previous publications should be properly cited and discussed, RNAseq results should be validated, and the categorization into "aggressive/not aggressive" breast cancers should be discussed as well.

If you feel you can satisfactorily address these points and those listed by the referees, you may wish to submit a revised version of your manuscript. Please attach a covering letter giving details of the way in which you have handled each of the points raised by the referees. A revised manuscript will once again be subject to review and we cannot guarantee at this stage that the eventual outcome will be favorable. EMBO Molecular Medicine encourages a single round of revision only and therefore, acceptance or rejection of the manuscript will depend on the completeness of your responses included in the next, final version of the manuscript. For this reason, and to save you from any frustrations in the end, I would strongly advise against returning an incomplete revision.

If you would like to discuss further the points raised by the referees, I am available to do so via email or video. Let me know if you are interested in this option.

Revised manuscripts should be submitted within three months of a request for revision; they will otherwise be treated as new submissions, except under exceptional circumstances in which a short extension is obtained from the editor.

When submitting your revised manuscript, please carefully review the instructions that follow below. We perform an initial quality control of all revised manuscripts before re-review; failure to include requested items will delay the evaluation of your revision.

We require:

1) A .docx formatted version of the manuscript text (including legends for main figures, EV figures and tables). Please make sure that the changes are highlighted to be clearly visible.

2) Individual production quality figure files as .eps, .tif, .jpg (one file per figure). For guidance, download the 'Figure Guide PDF' (https://www.embopress.org/page/journal/17574684/authorguide#figureformat).

3) At EMBO Press we ask authors to provide source data for the main figures. Our source data coordinator will contact you to discuss which figure panels we would need source data for and will also provide you with helpful tips on how to upload and organize the files.

4) A .docx formatted letter INCLUDING the reviewers' reports and your detailed point-by-point responses to their comments. As part of the EMBO Press transparent editorial process, the point-by-point response is part of the Review Process File (RPF), which will be published alongside your paper.

5) A complete author checklist, which you can download from our author guidelines (https://www.embopress.org/page/journal/17574684/authorguide#submissionofrevisions). Please insert information in the checklist that is also reflected in the manuscript. The completed author checklist will also be part of the RPF.

6) Please note that all corresponding authors are required to supply an ORCID ID for their name upon submission of a revised manuscript.

7) It is mandatory to include a 'Data Availability' section after the Materials and Methods. Before submitting your revision, primary datasets produced in this study need to be deposited in an appropriate public database, and the accession numbers and database listed under 'Data Availability'. Please remember to provide a reviewer password if the datasets are not yet public (see https://www.embopress.org/page/journal/17574684/authorguide#dataavailability).
In case you have no data that requires deposition in a public database, please state so in this section (This study includes no data deposited in external repositories). Note that the Data Availability Section is restricted to new primary data that are part of this study.

8) For data quantification: please specify the name of the statistical test used to generate error bars and P values, the number (n) of independent experiments (specify technical or biological replicates) underlying each data point and the test used to calculate p-values in each figure legend. The figure legends should contain a basic description of n, P and the test applied. Graphs must include a description of the bars and the error bars (s.d., s.e.m.). Please provide exact p values.

9) Our journal encourages inclusion of *data citations in the reference list* to directly cite datasets that were re-used and obtained from public databases. Data citations in the article text are distinct from normal bibliographical citations and should directly link to the database records from which the data can be accessed. In the main text, data citations are formatted as follows: "Data ref: Smith et al, 2001" or "Data ref: NCBI Sequence Read Archive PRJNA342805, 2017". In the Reference list, data citations must be labeled with "[DATASET]". A data reference must provide the database name, accession number/identifiers and a resolvable link to the landing page from which the data can be accessed at the end of the reference. Further instructions are available at .

10) We replaced Supplementary Information with Expanded View (EV) Figures and Tables that are collapsible/expandable online. A maximum of 5 EV Figures can be typeset. EV Figures should be cited as 'Figure EV1, Figure EV2" etc... in the text and their respective legends should be included in the main text after the legends of regular figures.
- For the figures that you do NOT wish to display as Expanded View figures, they should be bundled together with their legends in a single PDF file called *Appendix*, which should start with a short Table of Content. Appendix figures should be referred to in the main text as: "Appendix Figure S1, Appendix Figure S2" etc.
- Additional Tables/Datasets should be labeled and referred to as Table EV1, Dataset EV1, etc. Legends have to be provided in a separate tab in case of .xls files. Alternatively, the legend can be supplied as a separate text file (README) and zipped together with the Table/Dataset file.
See detailed instructions here:

11) The paper explained: EMBO Molecular Medicine articles are accompanied by a summary of the articles to emphasize the major findings in the paper and their medical implications for the non-specialist reader. Please provide a draft summary of your article highlighting
- the medical issue you are addressing,
- the results obtained and
- their clinical impact.
This may be edited to ensure that readers understand the significance and context of the research. Please refer to any of our published articles for an example.

12) For more information: There is space at the end of each article to list relevant web links for further consultation by our readers. Could you identify some relevant ones and provide such information as well? Some examples are patient associations, relevant databases, OMIM/proteins/genes links, author's websites, etc...

13) Author contributions: CRediT has replaced the traditional author contributions section because it offers a systematic machine readable author contributions format that allows for more effective research assessment. Please remove the Authors Contributions from the manuscript and use the free text boxes beneath each contributing author's name in our system to add specific details on the author's contribution. More information is available in our guide to authors.

14) Disclosure statement and competing interests: We updated our journal's competing interests policy in January 2022 and request authors to consider both actual and perceived competing interests. Please review the policy https://www.embopress.org/competing-interests and update your competing interests if necessary.

15) Every published paper now includes a 'Synopsis' to further enhance discoverability. Synopses are displayed on the journal webpage and are freely accessible to all readers. They include a short stand first (maximum of 300 characters, including space) as well as 2-5 one-sentences bullet points that summarizes the paper. Please write the bullet points to summarize the key NEW findings. They should be designed to be complementary to the abstract - i.e. not repeat the same text. We encourage inclusion of key acronyms and quantitative information (maximum of 30 words / bullet point). Please use the passive voice. Please attach these in a separate file or send them by email, we will incorporate them accordingly.

Please also suggest a striking image or visual abstract to illustrate your article as a PNG file 550 px wide x 300-600 px high.

16) As part of the EMBO Publications transparent editorial process initiative (see our Editorial at http://embomolmed.embopress.org/content/2/9/329), EMBO Molecular Medicine will publish online a Review Process File (RPF) to accompany accepted manuscripts.
In the event of acceptance, this file will be published in conjunction with your paper and will include the anonymous referee reports, your point-by-point response and all pertinent correspondence relating to the manuscript. Let us know whether you agree with the publication of the RPF and as here, if you want to remove or not any figures from it prior to publication.
Please note that the Authors checklist will be published at the end of the RPF.

EMBO Molecular Medicine has a "scooping protection" policy, whereby similar findings that are published by others during review or revision are not a criterion for rejection. Should you decide to submit a revised version, I do ask that you get in touch after three months if you have not completed it, to update us on the status.

I look forward to receiving your revised manuscript.

Yours sincerely,

Lise Roth

Lise Roth, PhD
Senior Editor
EMBO Molecular Medicine

***** Reviewer's comments *****

Referee #2 (Comments on Novelty/Model System for Author):

I should reveal that I'm not qualified to evaluate the statistical analyses and bioinformatic approaches.

Referee #2 (Remarks for Author):

This manuscript by Prekovic et al. reports a potential interaction between glucocorticoid and estrogen signaling in determining the response to chemotherapy in breast cancer models, specifically for subtypes (luminal A versus B).
The authors use cancer cell lines, xenograft models, tumor profiles and organoids together with NGS analyses (RNA profiling, ChIP) and proteomics (interactomes, protein expression) to find that the transcriptional crosstalk of these two steroid receptors may be targetable by drugs for epigenetic regulators.

This story has a high clinical relevance, and the omics techniques are state of the art. While the details of the molecular mechanism, besides ZBTB16 regulation, could be dissected more thoroughly, the potential impact and novelty would make this manuscript suitable for publication in EMBO Mol Med after the following points have been addressed:

- The authors perform RIME for GR and ER in two cell lines MCF-7 (luminal A) and EFM-192A (luminal B) and identify ER as a differential interactor of the GR. However it is unclear whether this is a general feature of luminal A and B subtypes or specific to these cell lines. Also, it should be compared how strongly ER is expressed in these different cell lines and if the interaction might be expression level - dependent.

- Also, a lot of the conclusions and findings are based on RNAseq profiles. I would recommend to include qRT-PCRs or Western Blots, Co-IPs... to validate these findings.

- The authors write 'Importantly, using HOMER motif analysis (Heinz et al, 2010) glucocorticoid binding motifs (GRE) were detected in ER binding regions to which GR also binds (Expanded View Fig. 3e), suggesting that co-binding does not occur via tethering, contrasting a prior report (Yang et al, 2017a).'
There are numerous reports on GR-DNA or GR-TF interactions in the literature which might actually support this observation, and which could be discussed here.
On that note, the bioinformatic motif analyses could be presented in more detail in the main figures. Why is this specific subset of ER target genes remodeled by GCs? Would a mutation of the binding site in the ZBTB16 locus abolish regulation? How about a reporter assay?

- The authors first describe a GR-ER crosstalk that is specific for luminal A type breast cancer. Then they identify common regulation of ZBTB16 as a GR/ER target that can suppress tumor growth. Later they are successful in treating both luminal A and luminal B models with epigenetic inhibitors.
Here, the storyline about how these findings are connected and mechanistically linked is hard to follow. Wouldn't it be expected

that inhibitors of epigenetic mechanisms generally inhibit tumor growth? Also, is their impact on cell survival specific to tumor cells or does it also impact healthy tissue? Are these effects GR-dependent, would they be lost in GR knockout cells or by co-treatment with RU486?

- A minor point, but Fig 1A is rather vague and schematic. A supplementary figure with the actual pipeline, filters, cutoffs... used to identify the GR signature would be more useful.

Referee #3 (Comments on Novelty/Model System for Author):

Very complex statistical modelling is used, with visualisation, but at times it is hard to follow, and to understand how false discovery risks are managed.
Models seem good, but in Fig 4 the organoids are not explained, legends and labels are poor.

Referee #3 (Remarks for Author):

This is a far-reaching and ambitious attempt to find new therapeutic avenues for breast cancer.

The authors are expert, and make good use of publicly available data, and generate significant new data using cutting edge approaches.

The scope if very broad, and at times it is hard for the reader to follow. Some additional erxplanation in the results section, better legends and labels would massively help.

In general the reader would be helped by additional care in the results section ewxplaining what was done and shown in the figs, and what the likely conclusions may be, with appropriate mention of alternative explanations and caveats. This is especially important as the data visualisation makes it impossible to determine what was done eg Fig 1c is totally incomprehensible, and comes with no explanation.

The discovery of ZBTB16 is interesting, but there is no mention of what it is, or its role in cell cycle progression. ZBTB16 is genetically associated with human phenotypes, and this might have been of interest. ZBTB16 gene re-arrangements are reported in leukemia, are they seen in breast cancers? The lack of curiousity about ZBTB16 is strange, as it looks like an interesting new avenue.

The final part relating to epifenetic regulators seems disconnected from the rest of the paper which leads to the discovery and exploration of ZBTB16 in breast cancer.

Specific points...

1 Fig 1c needs some work to make it useful. I could not see anything here.

2 Fig 1e this is helpful, but better labels and legend would help the reader to understand what was done, and what the authors think it shows them.

3 Fig 2F the PCA is helpful, but quite hard to see the two clusters, can the symbols be enlarged, or made more distinctive.

4 Fig 3c I could not understand what was done, or what this visualisation is supposed to be. It looks to me to be the opposite of what the authors claim in the text? This really needs attention.

5 Fig 3d the differences between some of the clusters is very small, how were peaks called, and how confident are they that ER/GR Dexa shared and ER Veh/Dex shared are different, they look ther same to me.

6 Fig 3f where is ZBTB16 on this plot? should be top right quadrant??

7 Fig 3h why does ER bind in response to Dex, in the absence of E2? is there E2 in the medium? Does ER stimulate ZBTB16 expression at mRNA and protein level, is that also seen with GR, how do the two NHRs interact in mediating this regulation? Is the GR efect solely through ZBTB16, or how much of the GR effect is through ZBTB16?

8 Fig 3j here its unclear if Dexa has an effect in the absence of over expressed ZBTB16, the group is not included, and does dexa alter the ZBTB16 effect?

9 Fig 4e i could not follow what was done, or what the dotted vertical line indicated. The statistical analysis is obscure, and it appears that nothing had any significant effect? is it appropriate to refer to eradication of ther cancer cells?

10 In the discussion the role of ZBTB16 again comes into question. If ER drives expression why does ER expression associate with worse prognosis?

11 The discussion point referring to ER and GR regulating concludes that ZBTB16 may be part of the answer, but this pathway is not fully explored.

12 I dont think they can claim the HDACi drives a similar pattern of gene expression at GR activation, I think this is based on Fig 4A but that is looking at GO terms of GR regulated genes, and not comparing Dexa and the HDACi.

Referee #4 (Comments on Novelty/Model System for Author):

See below. If other reviewers are more positive I would be happy to see a revision. I have 'conceptual' issues here, notwithstanding this publication which was 'surprisingly' not discussed:
https://pubmed.ncbi.nlm.nih.gov/32517789/

Referee #4 (Remarks for Author):

In this study, Prekovic et al show that the activity status of GR determines luminal subtype identity and has implications for patients' outcomes and they then go on to show that GR suppresses tumor growth by engaging with estrogen receptor (ER), leading to redistribution of ER on chromatin and upregulation of the ZBTB16 gene. Notably, they identify ZBTB16 as a hormone-driven inhibitor of ER-positive breast cancer growth. They demonstrate that clinically available epigenetic inhibitors, which mimic GR-induced gene repression, can effectively suppress the growth of highly aggressive ER-positive breast cancer cells displaying absence of GR activity. I have the following concerns:
At a general level, I have issues with the categorisation of aggressive and not aggressive based on the luminal type classification, and further the categorisation of 'highly aggressive cell lines'. I do not agree with this, and the lack of translational significance has been born out of the fact that the compounds used (eg. vorinistat, Panobinostat, in contrast to ribociclib for example, lack activity in patients - as does dexamethasone - as they know). In short, clinically available epigenetic inhibitors, which mimic GR-induced gene repression, whilst effectively suppress ER-positive cell growth, do not have benefits to patients thus far.
There is a mix of bioinformatics and wet lab work, but the former is used almost to hide the limited work of the latter and the mix of original data vs METABRIC mining for example is unclear.
This recent publication on ZBTB16 is not mentioned covering large aspects of the wet lab work and epigenetics:
https://pubmed.ncbi.nlm.nih.gov/32517789/
The organoid model work is the strongest aspect, but there is a lack of mechanism with knock in and knock out (siRNA and CRSPR) of the GR in ER+ or negative cell lines to validate their results, with corresponding in vivo experiments one would expect here.

Rebuttal Letter, Prekovic et al.

Referee #2 (Comments on Novelty/Model System for Author):

I should reveal that I'm not qualified to evaluate the statistical analyses and bioinformatic approaches.

Referee #2 (Remarks for Author):

This manuscript by Prekovic et al. reports a potential interaction between glucocorticoid and estrogen signaling in determining the response to chemotherapy in breast cancer models, specifically for subtypes (luminal A versus B). The authors use cancer cell lines, xenograft models, tumor profiles and organoids together with NGS analyses (RNA profiling, ChIP) and proteomics (interactomes, protein expression) to find that the transcriptional crosstalk of these two steroid receptors may be targetable by drugs for epigenetic regulators. This story has a high clinical relevance, and the omics techniques are state of the art. While the details of the molecular mechanism, besides ZBTB16 regulation, could be dissected more thoroughly, the potential impact and novelty would make this manuscript suitable for publication in EMBO Mol Med after the following points have been addressed:

We would like to express our gratitude to the reviewer for their insightful feedback on our manuscript. We appreciate the recognition of the high clinical relevance and state-of-the-art omics techniques used in our study on the potential interaction between glucocorticoid and estrogen signaling in breast cancer.

- The authors perform RIME for GR and ER in two cell lines MCF-7 (luminal A) and EFM-192A (luminal B) and identify ER as a differential interactor of the GR. However it is unclear whether this is a general feature of luminal A and B subtypes or specific to these cell lines. Also, it should be compared how strongly ER is expressed in these different cell lines and if the interaction might be expression level - dependent.

We greatly appreciate this comment of the reviewer and have now expanded our study to include GR RIME experiments in the ZR75-1 cell line, another Luminal A model. We observed an interaction between GR and ER in this model as well (**now included as Figure EV4d**). As for Luminal B cell lines, our laboratory has access to EFM-192A, in which we observed full nuclear translocation of GR upon activation (**as depicted in Figure EV4b; quantification of nuclear translocation**). Interestingly, for the other luminal B-derived cell lines, nuclear translocation of GR following its activation was less apparent (**Figure EV4b**). While interesting and precluding ER/GR crosstalk (as ER is predominantly in the nucleus), RIME in the additional Luminal B cell lines would also not show ER/GR interactions, but this would be due to differential subcellular localization. For this reason, performing RIME or any other downstream analysis in these would not be considered a fair comparison. We have added a section to the discussion that addresses these points and further elaborates on diverse mechanisms of GR inactivation. The segment is as follows:

"In relation to loss of GR activity, prior research has shown that GR may be inactivated through diverse mechanisms, including alterations in nuclear translocation capacity (Matthews et al, 2004) and co-regulator recruitment (Prekovic et al., 2021). In context of breast cancer, NR3C1 promotor methylation (Nesset et al, 2014), has been suggested to play a role in its transcriptional silencing, however other possible mechanisms of inactivation may also exist and should be dissected in further studies.

We agree with the reviewer that ER/GR levels are an important factor to consider. To address this issue, we have now included Western blot analyses for ER and GR across all models used in the manuscript (**Figure EV6c**).

- Also, a lot of the conclusions and findings are based on RNAseq profiles. I would recommend to include qRT-PCRs or Western Blots, Co-IPs... to validate these findings.

We appreciate the reviewer's suggestion to include additional validation methods. Since many of the analyses presented in the study were based on large public datasets such as the TCGA, additional experiments would not be possible for these samples. However, in addressing the reviewer comments, we now implemented the Reverse Phase Protein Array (RPPA) data from the TCGA cohort **(Figure EV2j)**, fully confirming our mRNA-based observations. To further validate and confirm the use of our signature by orthogonal methods, we in addition implemented the publicly

available single-cell RNA sequencing data from T47D cell line (**Figure EV3c-d**). The GR activity score perfectly separates the single cells based on the treatment time point, and we detect ZBTB16 expression in almost every cell following 18h treatment with Dexamethasone (**Figure EV4i**).
For ZBTB16 itself, sadly suitable antibodies are not available (*it has been elaborated by others, and also re-tested in our hands, that commercial ZBTB16 antibodies do not work – see Mao, Ai-Ping, et al, Proceedings of the National Academy of Sciences 113.27 (2016): 7602-7607*). In the revised version, the orthogonal approaches, and additional models, as explained above, have been added to substantiate our claims.
We hope that the reviewer will appreciate our use of diverse models (6 cell lines), organoids (7 models), MIND mouse models, as well as use of various clinical datasets, and cross-validation performed between different omic-techniques.

- The authors write 'Importantly, using HOMER motif analysis (Heinz et al, 2010) glucocorticoid binding motifs (GRE) were detected in ER binding regions to which GR also binds (Expanded View Fig. 3e), suggesting that co-binding does not occur via tethering, contrasting a prior report (Yang et al, 2017a).' There are numerous reports on GR-DNA or GR-TF interactions in the literature which might actually support this observation, and which could be discussed here. On that note, the bioinformatic motif analyses could be presented in more detail in the main figures. Why is this specific subset of ER target genes remodeled by GCs? Would a mutation of the binding site in the ZBTB16 locus abolish regulation? How about a reporter assay?

We thank the reviewer for this comment. While we agree that various reports in literature exist, there has been quite some discussion in the field if especially in terms of ER and GR the interaction occurs via tethering. Our experiments showing that degradation of ER by use of fulvestrant does not reduce GR binding to the genome (**Figure EV4g**), is contrasting these studies, and rather support a model of direct GR-chromatin binding. We have now included a discussion point on these observations and the disparities with 'tethering model literature' – highlighting that we are only focusing on ER/GR interactions and how they jointly regulate expression of genes -including ZBTB16- in a tethering-independent fashion.

"Nonetheless, loss of GR action seems to be intricately associated with the uncoupling of GR from ER. The crosstalk between nuclear receptors may be one of the leading mechanisms that control proliferative capacity of breast cancer (Mayayo-Peralta et al, 2021a), However, it remains unclear how GR and ER interactions occur. It was previously suggested that co-occupancy of these nuclear receptors is due to tethering of GR to DNA-bound ER (Yang et al., 2017), resulting in repression of the ER-transcriptional program. Studies on human breast cancers (Severson et al, 2018), as well as our data, do not support this but rather suggest that GR binds in proximity of ER binding sites possibly competing with its binding to DNA. Future research should address the mechanistic underpinnings of GR-ER crosstalk, and particularly focus on how ER action is altered by GR activation."

Indeed, the re-location of ER is an interesting point, we have performed HOMER analysis on these sites now. However, in light of the newly rewritten manuscript we chose not to include it in as it doesn't impact or alter the conclusions of our manuscript. However, the analysis of top motifs can be seen in this rebuttal letter:

Rank	Motif	P-value	log P-pvalue	% of Targets	% of Background	STD(Bg STD)	Best Match/Details	Motif File
1	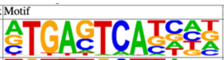	1e-111	-2.573e+02	32.06%	11.91%	122.9bp (182.8bp)	JunB(bZIP)/DendriticCells-Junb-ChIP-Seq(GSE36099)/Homer(0.993) More Information | Similar Motifs Found	motif file (matrix)
2	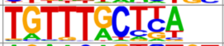	1e-93	-2.153e+02	37.83%	17.37%	135.8bp (179.6bp)	FOXM1(Forkhead)/MCF7-FOXM1-ChIP-Seq(GSE72977)/Homer(0.932) More Information | Similar Motifs Found	motif file (matrix)
3	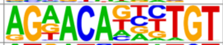	1e-78	-1.797e+02	17.00%	4.92%	104.3bp (181.0bp)	GRE(NR),IR3/RAW264.7-GRE-ChIP-Seq(Unpublished)/Homer(0.920) More Information | Similar Motifs Found	motif file (matrix)
4	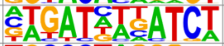	1e-56	-1.301e+02	17.33%	6.38%	119.7bp (180.4bp)	GATA(Zf),IR3/iTreg-Gata3-ChIP-Seq(GSE20898)/Homer(0.962) More Information | Similar Motifs Found	motif file (matrix)
5	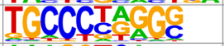	1e-47	-1.094e+02	44.28%	28.14%	150.3bp (181.9bp)	TFAP2B/MA0811.1/Jaspar(0.887) More Information | Similar Motifs Found	motif file (matrix)
6	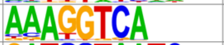	1e-46	-1.066e+02	40.78%	25.27%	137.0bp (183.4bp)	NR4A1/MA1112.2/Jaspar(0.989) More Information | Similar Motifs Found	motif file (matrix)
7	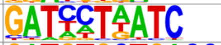	1e-41	-9.512e+01	13.00%	4.81%	142.3bp (180.2bp)	GATA(Zf),IR4/iTreg-Gata3-ChIP-Seq(GSE20898)/Homer(0.840) More Information | Similar Motifs Found	motif file (matrix)
8	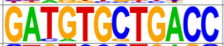	1e-23	-5.376e+01	0.61%	0.00%	67.3bp (0.0bp)	PB0041.1_Mafb_1/Jaspar(0.760) More Information | Similar Motifs Found	motif file (matrix)
9	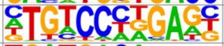	1e-22	-5.287e+01	19.61%	11.46%	141.5bp (183.3bp)	Nr5a2/MA0505.1/Jaspar(0.754) More Information | Similar Motifs Found	motif file (matrix)
10	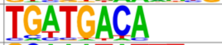	1e-22	-5.132e+01	31.28%	21.33%	151.9bp (184.1bp)	Tbx20(T-box)/Heart-Tbx20-ChIP-Seq(GSE29636)/Homer(0.877) More Information | Similar Motifs Found	motif file (matrix)
11	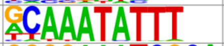	1e-19	-4.378e+01	11.94%	6.20%	158.4bp (172.0bp)	Arid5a/MA0602.1/Jaspar(0.789) More Information | Similar Motifs Found	motif file (matrix)
12	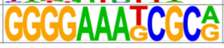	1e-18	-4.246e+01	0.50%	0.00%	68.3bp (84.1bp)	RELB/MA1117.1/Jaspar(0.702) More Information | Similar Motifs Found	motif file (matrix)

We agree with the reviewer that further genetic dissection of the enhancer would have been fantastic, during the last 3 months of the revision period we have tried to perform CRISPR-mediated disruptions of the GREs in the enhancer but ran into technical difficulties, unfortunately. We have now acknowledged that in the new version of the manuscript, acknowledging the limitations and future points to be addressed.

"We next focused on genomic regions to which we detected ER binding solely upon GR activation (Fig. 3e; n=1803 sites). Utilizing Cistrome-GO (Li et al, 2019) we functionally annotated these sites to individual genes, yielding a score that represents the likelihood of regulation (i.e. Regulatory Potential (RP)). By relating the RP to differential expression of genes following Dexa-treatment, we identified ZBTB16 gene as a potential driver of Dexa-induced growth suppression (Fig. 3g). As seen from the binding profiles, ER is actively occupying the ZBTB16 intron sites only once GR is activated and bound at overlapping locations (Fig. 3h). Upregulation of ZBTB16 expression was not observed when activating ER in absence of glucocorticoid (Expanded View Fig. 4j). Whether ER transcriptional activity is necessary for upregulation of ZBTB16 expression, or whether ER only acts as modulator of GR-driven upregulation of ZBTB16, remains unknown and should be address by future studies. Importantly, the glucocorticoid-induced upregulation of ZBTB16 is not exclusive for MCF-7, and is also seen in T47D cells; another model derived from a luminal A cancer (scRNA sequencing time-course; Expanded View Fig. 4i)."

The authors first describe a GR-ER crosstalk that is specific for luminal A type breast cancer. Then they identify common regulation of ZBTB16 as a GR/ER target that can suppress tumor growth. Later they are successful in treating both luminal A and luminal B models with epigenetic inhibitors. Here, the storyline about how these findings are connected and mechanistically linked is hard to follow. Wouldn't it be expected that inhibitors of epigenetic mechanisms generally inhibit tumor growth? Also, is their impact on cell survival specific to tumor cells or does it also impact healthy tissue? Are these effects GR-dependent, would they be lost in GR knockout cells or by co-treatment with RU486?

Indeed, we agree with the reviewer these inhibitors should generally affect growth, we would propose that cancers without active GR signaling would be more sensitive to these inhibitors. In our models, absence of GR activation is equal to GR-KO (as described in our previous study in lung cancer *(Prekovic et al., 2021 Nat. Com)*). We have now tried to better explain the reasoning and provide a better link to the epigenome findings. Firstly, we now cite the articles related to *ZBTB16* and its role in altering the epigenome and state that we pursue alternative therapeutic strategies as *ZBTB16* cannot be therapeutically utilized, and secondly, we expand our findings showing activation of GR leads to changes in the epigenome (we performed ChIP for H3K27Ac following long-term exposure to glucocorticoids), in close proximity to genes involved in cancer proliferation and migration (**Figure 4c-d** and **Figure EV6b**). These data give additional support to our hypothesis that loss of GR activity leads to epigenetic changes that drive aggressiveness in breast cancer.

- A minor point, but Fig 1A is rather vague and schematic. A supplementary figure with the actual pipeline, filters, cutoffs… used to identify the GR signature would be more useful.

We thank the reviewer for highlighting this unclarity. Following the reviewer's advance, we have included a supplementary figure (**Figure EV1**) depicting all the important parts in our analysis, providing deeper insights and more details on the filters, cutoffs and general approach taken.

Referee #3 (Comments on Novelty/Model System for Author):

Very complex statistical modelling is used, with visualisation, but at times it is hard to follow, and to understand how false discovery risks are managed.

We thank the reviewer for this comment – in line with this we have now improved these aspects of the manuscript, better explaining how all analyses were performed. Particularly, we have included the new **Figure EV1** that summarizes how GR activity signature was developed. Additionally, in the captions of the figures statistical tests are now mentioned, and for particular bioinformatic explorations we have used random-gene iterations which are a standard in the field of genomics. For all of the omic experiments we have done statistical testing and the p-values were corrected for multiple testing using Benjamini and Hochberg method, thus managing the false discovery risks. This is now mentioned

throughout the amnsucript. In addition, we have used orthogonal approaches to validate our findings across different models.

Models seem good, but in Fig 4 the organoids are not explained, legends and labels are poor.

We thank the reviewer for highlighting this – we have now improved these figures, adding cartoons depicting models and highlighting the differences, additionally the statistical analysis was added (see below). Labels have also been improved.

Referee #3 (Remarks for Author):

This is a far-reaching and ambitious attempt to find new therapeutic avenues for breast cancer. The authors are expert, and make good use of publicly available data, and generate significant new data using cutting edge approaches.

We thank the reviewer for this nice comment.

The scope if very broad, and at times it is hard for the reader to follow. Some additional erxplanation in the results section, better legends and labels would massively help.

In agreement with all the comments of the reviewers we have extensively worked on revising the text and making the manuscript more coherent – we hope that the reviewer will find that parts of the manuscript are now better connected, and easier to read and comprehend.

In general the reader would be helped by additional care in the results section ewxplaining what was done and shown in the figs, and what the likely conclusions may be, with appropriate mention of alternative explanations and caveats. This is especially important as the data visualisation makes it impossible to determine what was done eg Fig 1c is totally incomprehensible, and comes with no explanation.

We apologize for this unclarity. Following the reviewers' recommendations, we have now improved visualization and explained our findings / figures better. Additionally, for each figure we tried to provide schematics representation when applicable to improve readability. Additionally, we have now added parts throughout the manuscript stating what we have not addressed, the caveats and what the future perspectives could be.

The discovery of ZBTB16 is interesting, but there is no mention of what it is, or its role in cell cycle progression. ZBTB16 is genetically associated with human phenotypes, and this might have been of interest. ZBTB16 gene re-arrangements are reported in leukemia, are they seen in breast cancers? The lack of curiousity about ZBTB16 is strange, as it looks like an interesting new avenue.

We absolutely agree with the reviewer, and we are currently working on *ZBTB16* in follow-up studies to filly understand the complex action of ZBTB16. However, given its complex mode-of-action, we respectfully feel that this goes beyond the scope of the current manuscript, but warrant a separate study by itself (also not to overload the current manuscript). To addres the reviewer's concern, we have however now expended the part of the results and discussion sections focused on *ZBTB16*. Importantly, to go deeper in the molecular action of ***ZBTB16***, we also performed RIME analysis on V5-tagged *ZBTB16* in MCF7 cell line (**Figure EV5k-i**). We now propose that it may alter NR signaling through interaction with various NR coregulators. Thereby providing novel avenues for further research in context of *ZBTB16* in ER+ breast cancer and also provides a potential link between ZBTB16 and epigenetic regulation by GR (see below), as mentioned in our revised manuscript.

The final part relating to epifenetic regulators seems disconnected from the rest of the paper which leads to the discovery and exploration of ZBTB16 in breast cancer.

We appreciate that the reviewer pointing this out. As further elaborated in the response to point 12 raised by this reviewer, our reasoning was the following: As GR activation -which diminishes cell viability in Luminal A breast cancer- leads to a downregulation of genes involved in epigenetic regulation and epigenetic remodeling, we hypothesized that inhibitors for these proteins would effectively also

decrease proliferation, phenocopying the cellular response to GR activation, also in cells in which GR was not active. The inhibitors used, were selected as proof-of-concept representatives of this inhibitor class. This line-of-reasoning is now better explained in the discussion section. We now also reflect on the ZBTB16 axis not having therapeutic potential by itself – and that thus we explored alternative means to 'bypass' the GR-activation cascade. Curiously, as elaborated above, in our newly added RIME data we see that ZBTB16 interacts with various transcriptional repressors (e.g. NCORs) and that GR activation leads to loss of H3K27Ac at particular sites in the genome that may regulate cancer-driving genes. While we do not formally address if this is driven solely by ZBTB16, we do include a segment in the manuscript hypothesizing this.

1 Fig 1c needs some work to make it useful. I could not see anything here.

We thank the reviewer for bringing this to our attention. Figure 1c can now be seen as **Figure EV2i** and updated the caption to aid interpretation. The idea behind this panel was to show that no single tumour type drives our findings. To improve the quality and interpretation of the data, in the revised version of the manuscript, Figure 1c has now been replaced by a snake plot showing the correlation score for each of the pathways with GR activity (**Figure 1c**). We highlighted some of the pathways and included scatter plots of these individual pathways displaying their relationship with GR activity across all the samples. We believe that this better illustrates that GRa is correlating with specific pathways (and not with all pathways), and that some of these have previously been reported by others, providing internal controls for our analyses but also crediting the work of others better.

2 Fig 1e this is helpful, but better labels and legend would help the reader to understand what was done, and what the authors think it shows them.

We have now improved this panel, and provided additional details in the caption, to further improve clarity.

3 Fig 2F the PCA is helpful, but quite hard to see the two clusters, can the symbols be enlarged, or made more distinctive.

We have now increased the size of the circles plotted we hope that the reviewer finds the figure easier to read and that the clusters are more apparent.

4 Fig 3c I could not understand what was done, or what this visualisation is supposed to be. It looks to me to be the opposite of what the authors claim in the text? This really needs attention.

We thank the reviewer for highlighting this unclarity. We have now explained it better; the visualization is truly depended on how the conditions are labeled / placed (MCF-7 vs EFM or the other way around). We have now changed the order of the conditions in our script therefore the curve is positive, and more logically aligns with the conclusions.

5 Fig 3d the differences between some of the clusters is very small, how were peaks called, and how confident are they that ER/GR Dexa shared and ER Veh/Dex shared are different, they look ther same to me.

All of our ChIP analysis is done with multiple replicates and according to the standards of the field. We have used standard pipelines for ChIP-seq and performed peak-calling with the most-recent version of MACS (MACS 3.0). Then the consensus analysis was done by mspc tool, to prioritize the peaks that are robustly called over replicate experiments, providing high confidence in our analysis.

6 Fig 3f where is ZBTB16 on this plot? should be top right quadrant??

Since *ZBTB16* is not an annotated ER target it is not found in this particular figure that only plots annotated ER targets (Estrogen receptor early; Hallmarks gene-set). Gene expression changes induced by glucocorticoids in MCF-7 cells can be seen in **Figure 3g**. Additionally, as described below, using E2 stimulation of cells grown in serum-striped conditions we show that E2 by itself doesn't alter *ZBTB16* expression (**Figure EV4j**) – explaining that indeed it should not be found in this particular figure.

7 Fig 3h why does ER bind in response to Dex, in the absence of E2? is there E2 in the medium? Does ER stimulate ZBTB16 expression at mRNA and protein level, is that also seen with GR, how do the two NHRs interact in mediating this regulation? Is the GR efect solely through ZBTB16, or how much of the GR effect is through ZBTB16?

We apologies for the confusion. For these experiments we use full growth conditions in which ER is active (indeed E2 is present in the medium). ER activation itself, without GR activity, is not enough to upregulate ZBTB16 (also seen in the newly added panel – **Figure EV4j**). We only see *ZBTB16* being regulated when Dexamethasone is added to the medium. The question of how much of the effect of GR is through ZBTB16 is an important and interesting one and is something we are currently trying to understand in our lab, in follow-up studies. We have now included parts in the manuscript discussing this and also reflecting on this in terms of future directions. Additionally, we tone down some of the conclusions made in our article. Interestingly, from the ZBTB16-RIME data that we added to the revised manuscript, this protein seems to interact with various transcription factors, this may, indeed, be novel way nuclear receptors indirectly affect gene expression beyond the classical-direct regulation.

8 Fig 3j here its unclear if Dexa has an effect in the absence of over expressed ZBTB16, the group is not included, and does dexa alter the ZBTB16 effect?

We thank the reviewer for this comment. For the ZBTB16-OE group we never performed treatment with Dexa, as the reviewer pointed out, this would definitely be of interest and it would have required us to repeat the experiments again, which would be hard to do in respect to animal ethics committee at our institute. Additionally, these experiments lasted 8 months therefore we wouldn't have had enough time to do them for this revision.

9 Fig 4e i could not follow what was done, or what the dotted vertical line indicated. The statistical analysis is obscure, and it appears that nothing had any significant effect? is it appropriate to refer to eradication of ther cancer cells?

We thank the reviewer for this comment and for noticing this being omitted. We have indeed not included statistical analysis of this in the original version. Now we have performed this and included it in the figure (also for organoid models found in **Fig. EV6h**). The dotted line was also removed as it was introducing confusion. Additionally, to help the figure interpretation we have included schemes representing organoid models above the panels. We also note that we were looking at viability and not cancer cell eradication with our readouts, throughout the manuscript.

10 In the discussion the role of ZBTB16 again comes into question. If ER drives expression why does ER expression associate with worse prognosis?

Its an interesting point, as we do not claim that ER alone drives expression, it is the combination of ER and active GR. Loss of GR action occurs in more aggressive disease, where regulation of ZBTB16 is also lost. We have now clarified this better in the discussion section. We also include RNA sequencing analysis of E2 effect on gene expression, following serum starvation. In these experiments it is clearly demonstrated that E2 activation of ER is not enough to induce ZBTB16 expression (**Figure EV4j**).

11 The discussion point referring to ER and GR regulating concludes that ZBTB16 may be part of the answer, but this pathway is not fully explored.

Indeed, as elaborated above, E2 does not activate ZBTB16 on its own (we have now included CSS + E2 experiments); only addition of GR agonists along with ER-activating ligands allows for regulation of ZBTB16 (Fig. EV4j). We have further expanded the part of the discussion reflecting on the regulation of ZBTB16.

12 I dont think they can claim the HDACi drives a similar pattern of gene expression at GR activation, I think this is based on Fig 4A but that is looking at GO terms of GR regulated genes, and not comparing Dexa and the HDACi.

We apologize for the confusion; indeed, this is not the claim that we wanted to convey. We have now amended the text to improve clarity; As GR activation -which diminishes cell viability in Luminal A breast cancer- leads to a downregulation of genes involved in epigenetic regulation and epigenetic remodelling, we hypothesized that inhibitors for these proteins would effectively also decrease proliferation, phenocopying the cellular response to GR activation, irrespective of GR activation status. The inhibitors used, were selected as proof-of-concept representatives of this inhibitor class. This line-of-reasoning is now better explained in the discussion section.

Referee #4 (Comments on Novelty/Model System for Author):

See below. If other reviewers are more positive I would be happy to see a revision. I have 'conceptual' issues here, notwithstanding this publication which was 'surprisingly' not discussed: https://pubmed.ncbi.nlm.nih.gov/32517789/

Thank you for pointing this out. We are aware of this publication; however we have not included it as the whole article focuses on ZBTB16 in triple negative breast cancer models – and lacks any context in terms of steroid-hormone receptor action. Nonetheless, we appreciate the reviewers input and have taken the opportunity to expand our list of cited works to include more comprehensive discussions on the tumor suppressive action of ZBTB16, not only in TNBC (the referenced work), but also in prostate cancer (Han et al, 2019) and lung cancer (Wang et al, 2013).

Referee #4 (Remarks for Author):

In this study, Prekovic et al show that the activity status of GR determines luminal subtype identity and has implications for patients' outcomes and they then go on to show that GR suppresses tumor growth by engaging with estrogen receptor (ER), leading to redistribution of ER on chromatin and upregulation of the ZBTB16 gene. Notably, they identify ZBTB16 as a hormone-driven inhibitor of ER-positive breast cancer growth. They demonstrate that clinically available epigenetic inhibitors, which mimic GR-induced gene repression, can effectively suppress the growth of highly aggressive ER-positive breast cancer cells displaying absence of GR activity. I have the following concerns:

At a general level, I have issues with the categorisation of aggressive and not aggressive based on the luminal type classification, and further the categorisation of 'highly aggressive cell lines'. I do not agree with this, and the lack of translational significance has been born out of the fact that the compounds used (eg. vorinistat, Panobinostat, in contrast to ribociclib for example, lack activity in patients - as does dexamethasone - as they know). In short, clinically available epigenetic inhibitors, which mimic GR-induced gene repression, whilst effdectively suppress ER-positive cell growth, do not have benefits to patients thus far.
There is a mix of bioinformatics and wet lab work, but the former is used almost to hide the limited work of the latter and the mix of original data vs METABRIC mining for example is unclear.

We appreciate the reviewer's concerns regarding the categorization of aggressive and non-aggressive cell lines based on luminal type classification, as well as the limited translational significance of some compounds. In response, we have revised the manuscript to better emphasize that the luminal A/B distinction does not necessarily predict aggressiveness, but rather GR activity does. Additionally, we now refer to each of the models as derived from luminal A/B cancers. We also refrain from using the term ‘aggressiveness’ throughout the manuscript, and hope that the reviewer appreciates the changes we made in this respect.

We acknowledge the limited success of certain epigenetic inhibitors, such as vorinostat and panobinostat, and contrast their performance with ribociclib, an inhibitor that has shown promising results. This observation highlights the need for continued research and development of more effective therapeutics. Regarding dexamethasone, its effects have not been adequately evaluated in breast cancer clinical trials, making it difficult to draw definitive conclusions. We have added a section in the discussion to address these points:

“As we show here, activation of GR leads to changes in the H3K27Ac landscape of breast cancer cells. Of particular importance, glucocorticoid exposure leads to loss of H3K27Ac in vicinity of genes involved in proliferation and metastasis. Conversely, these sites are marked by H3K27Ac when GR is inactive

and are potentially of high importance in progression of breast cancer. Inspired by these findings, we used various epigenetic inhibitors (that block action of either HDACs or BRDs) to position these as promising therapeutic strategies for cancers that do not have an active GR signaling axis, such as luminal B breast cancers. Epigenetic inhibitors are utilized to treat hematological malignancies but their single agent-efficacy is yet to be established in solid cancers. The limited success of these treatments in solid cancers can potentially be attributed to patient group selection. Therefore, future studies in breast cancer, especially in luminal subtypes, may use GR activity as a biomarker but also utilize the new generation of epigenetic inhibitors, applied alone or in combination with standard-of-care therapeutics for breast cancer."

We respectfully disagree with the assertion that our manuscript attempts to hide any limitations in our experimental work. Our goal has always been to base our experiments on solid dry-lab foundations and to be transparent about our methods and findings. To address any concerns about the integration of bioinformatics and wet lab work, we have now clarified the relationship between our original data and the METABRIC dataset in the manuscript, ensuring that the contributions of each are clearly delineated.

We do want to stress, that while no study is perfect, we have used diverse experimental models (6 cell lines – 3 derived from luminal A and 3 derived from luminal B cancers), organoids (7 models), MIND mouse models (orthotopic engraftment of MCF-7 cells, overexpressing ZBTB16), as well as use of diverse clinical datasets (TCGA, metabric, KM cohort, etc.), and have performed various cross-validation using different omic-techniques.

For clarity and transparency, we list below all the publicly available data used in this study, as well as the one generated by us (and previously shown in the data-availability section through accessions code for both GEO and PRIDE repositories):

**Public data-sets used:**	**Generated by us for this manuscript:**
TCGA (mRNA and RPPA)	ChIP-seq data for ER and GR in MCF-7 cell line
KM plotter cohort	ChIP-seq data for H3K27Ac in MCF-7 cell line
METABRIC	RIME data for GR in MCF-7, EFM-192A and ZR-75-1
T47D scRNA seq	RIME data for ER in MCF-7 and EFM-192A
	RIME data for V5-tagged ZBTB16 in MCF-7 cell line
	Various proteomic experiments
	Various RNA sequencing data for different cell line models and conditions
	Organoid experiments with 7 lines, and various treatments
	MIND orthotopic mouse experiments with ZBTB16 over-expression
	MATADOR RNA seq
	Numerous *in vitro* experiments

We hope these revisions adequately address the reviewer's concerns and enhance the overall transparency quality of our work.

This recent publication on ZBTB16 is not mentioned covering large aspects of the wet lab work and epigenetics: https://pubmed.ncbi.nlm.nih.gov/32517789/

As elaborated above, we respectfully disagree with the reviewer on this aspect: the particular publication focuses on ZBTB16 in TNBC. As we focus on the ER positive disease, GR as a regulator of ZBTB16 expression and the epigenetic regulation thereof, we did not include the reference, as it doesn't address the main topic of our work: regulation of ZBTB16 in ER+ disease. In the new version of the manuscript, we do include this reference, in addition to other references that demonstrate that ZBTB16 is a tumor suppressor in various cancer types, as stated above.

The organoid model work is the strongest aspect, but there is a lack of mechanism with knock in and knock out (siRNA and CRSPR) of the GR in ER+ or negative cell lines to validate their results, with corresponding in vivo experiments one would expect here.

We appreciate the reviewer's recognition of the strengths in our organoid model work and their suggestion to perform knock-in and knock-out experiments using siRNA and CRISPR in ER+ or ER- cell lines to validate our results. However, it is important to note that, as demonstrated in our previous work (Prekovic et al, 2021) and corroborated by various other labs, knocking out GR has been shown to produce no significant effect in these models. As GR is a ligand-driven transcription factor, hormone action is the most-logical mode-of-action in this context. We hope this explanation addresses the reviewer's concerns and underscores the rationale for our chosen experimental approach.

Moreover, our current research demonstrates that GR is expressed in all the models we have investigated, as evident in the figures provided in the revised version of the manuscript (**Figure EV6c**). Given these observations, we believe that the suggested experiments would not significantly enhance our understanding of the underlying mechanisms, nor provide additional validation to our results. Instead, we have focused on other experimental approaches that are more pertinent and informative in the context of our study. We hope this explanation addresses the reviewer's concerns and clarifies our rationale for not including these specific experiments in our research.

13th Sep 2023

Dear Prof. Zwart,

Thank you for the submission of your revised manuscript to EMBO Molecular Medicine, and please accept my apologies for the delay in getting back to you, as one referee needed more time to complete his/her evaluation. We have now received the reports from the 3 referees who re-reviewed your manuscript. As you will see, they are supportive of publication, and we will therefore be able to accept your manuscript once the following editorial points will be addressed:

1/ Main manuscript file:
- Please address the queries from our data editors in the related Data Edited manuscript file. Kindly keep in track changes mode any new modification.
- Authors Merel Roest, Maliha Wajahat, Amy Dwyer, Wayne Tilley and Theresa Hickey were not included in the original submission, could you please clarify?
- Please provide up to 5 keywords.
- Please remove "Data not shown" (p.7). As per our guidelines on "Unpublished Data", the journal does not permit citation of "Data not shown". All data referred to in the paper should be displayed in the main or Expanded View figures.
- Materials and methods:
o Mice: please indicate the origin and gender of the mice used for the experiments.
o Statistics: please include statements about blinding, randomization, inclusion/exclusion criteria and adjust the author checklist accordingly.
- Thank you for providing a Data Availability section. Note that the Data Availability Section is restricted to new primary data that are part of this study. Please also remove "All the other data are available within the article and its Supplementary Information." We kindly remind you that datasets must be available to the public before publication of your manuscript.
- Acknowledgements: All funding sources should be added to the submission system, and match the information provided in the manuscript (currently missing in the submission system: Oncode Institute, NVKP_16-1-2016-0037 Breast Cancer Now Fellowship Award, Walk the Walk (2019-08-SF1310), Science Foundation Ireland (20/FFP-P/8597and the Dutch NWO X-omics Initiative are missing in EJP).
- Author contributions: CRediT has replaced the traditional author contributions section because it offers a systematic machine readable author contributions format that allows for more effective research assessment. Please remove the Authors Contributions from the manuscript and use the free text boxes beneath each contributing author's name in our system to add specific details on the author's contribution. More information is available in our guide to authors.
- Please rename the "conflict of interest" section to "Disclosure statement and competing interests". We updated our journal's competing interests policy in January 2022 and request authors to consider both actual and perceived competing interests. Please review the policy https://www.embopress.org/competing-interests and update your competing interests if necessary.

2/ Figures:
- We usually accommodate up to 5 EV figures. As you currently have 4 main figures and 6 EV figures, would you consider making 1 (or more) EV figure(s) a main figure? Please also rename the EV figures "Figure EV1", etc.
- Please add title and legend to your EV tables.
- Please make sure all figures and figure panels are correctly referenced in the text. There is currently a callout for Fig 4f but no such panel; callouts are missing for Fig EV3 F,G and there is a callout for Fig. EV3I but there is no such panel.
- Please carefully check the panels for Fig. EV2 and EV5: the legends, callouts and figure panels do not match.

3/ At EMBO Press we ask authors to provide source data for the main figures. Our source data coordinator will contact you to discuss which figure panels we would need source data for and will also provide you with helpful tips on how to upload and organize the files.

4/ Checklist:
- Please make sure that the statistic section matches the information provided in the manuscript.
- Please fill out the right section regarding sample definition and in-laboratory replication.

5/ The paper explained: EMBO Molecular Medicine articles are accompanied by a summary of the articles to emphasize the major findings in the paper and their medical implications for the non-specialist reader. Please provide a draft summary of your article highlighting
- the medical issue you are addressing,
- the results obtained and
- their clinical impact.
This may be edited to ensure that readers understand the significance and context of the research. Please refer to any of our published articles for an example.

6/ Every published paper now includes a 'Synopsis' to further enhance discoverability. Synopses are displayed on the journal webpage and are freely accessible to all readers. They include a short stand first (maximum of 300 characters, including space) as well as 2-5 one-sentences bullet points that summarizes the paper. Please write the bullet points to summarize the key NEW findings. They should be designed to be complementary to the abstract - i.e. not repeat the same text. We encourage inclusion of key acronyms and quantitative information (maximum of 30 words / bullet point). Please use the passive voice. Please attach these in a separate file or send them by email, we will incorporate them accordingly.

Please also suggest a striking image or visual abstract to illustrate your article as a PNG file 550 px wide x 300-600 px high.

7/ As part of the EMBO Publications transparent editorial process initiative (see our Editorial at http://embomolmed.embopress.org/content/2/9/329), EMBO Molecular Medicine will publish online a Review Process File (RPF) to accompany accepted manuscripts.
This file will be published in conjunction with your paper and will include the anonymous referee reports, your point-by-point response and all pertinent correspondence relating to the manuscript. Let us know whether you agree with the publication of the RPF and as here, if you want to remove or not any figures from it prior to publication.
Please note that the Authors checklist will be published at the end of the RPF.

I look forward to receiving your revised manuscript.

With kind regards,

Lise Roth

Lise Roth, PhD
Senior Editor
EMBO Molecular Medicine

***** Reviewer's comments *****

Referee #2 (Remarks for Author):

In this revised version, the authors have addressed most of my comments. Again, it would have been nice to see some more confirmatory, causative LOF experiments or protein levels, but I understand why this may not be feasible. I would at this point recommend publication of this improved manuscript.

Referee #3 (Comments on Novelty/Model System for Author):

Interesting, but very basic studies.

Referee #3 (Remarks for Author):

I think this is much improved. The clarity has been greatly enhanced. It remains quite preliminary, but interesting, and now it is comprehensible!

Referee #4 (Comments on Novelty/Model System for Author):

Very clear responses to my comments here

Referee #4 (Remarks for Author):

The authors have done a very good job in answering my comments which were not straightforward, and to my mind the other referees. Congratulations! Justin Stebbing

The authors addressed the minor editorial issues.

4th Oct 2023

Dear Prof. Zwart,

I am pleased to inform you that your manuscript is now accepted for publication and will be sent to our publisher to be included in the next available issue of EMBO Molecular Medicine!

Please note that I have added the mention "Figure legends" in the checklist (right column) for sample definition and in-laboratory replication. Please let us know immediately if you do not agree.

Congratulations on your interesting work!

With kind regards,

Lise Roth

Lise Roth, Ph.D
Senior Editor
EMBO Molecular Medicine

Follow us on Twitter @EmboMolMed
Sign up for eTOCs at embopress.org/alertsfeeds

*** *** *** IMPORTANT INFORMATION *** *** ***

SPEED OF PUBLICATION
The journal aims for rapid publication of papers, using using the advance online publication "Early View" to expedite the process: A properly copy-edited and formatted version will be published as "Early View" after the proofs have been corrected. Please help the Editors and publisher avoid delays by providing e-mail address(es), telephone and fax numbers at which author(s) can be contacted.

Should you be planning a Press Release on your article, please get in contact with embomolmed@wiley.com as early as possible, in order to coordinate publication and release dates.

LICENSE AND PAYMENT:

All articles published in EMBO Molecular Medicine are fully open access: immediately and freely available to read, download and share.

EMBO Molecular Medicine charges an article processing charge (APC) to cover the publication costs. You, as the corresponding author for this manuscript, should have already received a quote with the article processing fee separately. Please let us know in case this quote has not been received.

Once your article is at Wiley for editorial production you will receive an email from Wiley's Author Services system, which will ask you to log in and will present you with the publication license form for completion. Within the same system the publication fee can be paid by credit card, an invoice, pro forma invoice or purchase order can be requested.

Payment of the publication charge and the signed Open Access Agreement form must be received before the article can be published online.

PROOFS

You will receive the proofs by e-mail approximately 2 weeks after all relevant files have been sent o our Production Office. Please return them within 48 hours and if there should be any problems, please contact the production office at embopressproduction@wiley.com.

Please inform us if there is likely to be any difficulty in reaching you at the above address at that time. Failure to meet our deadlines may result in a delay of publication.

All further communications concerning your paper proofs should quote reference number EMM-2023-17737-V3 and be directed to the production office at embopressproduction@wiley.com.

EMBO Press Author Checklist

Corresponding Author Name: Stefan Prekovic and Wilbert Zwart	
Journal Submitted to: EMBO Molecular Medicine	
Manuscript Number: 2023-17737-V3	

USEFUL LINKS FOR COMPLETING THIS FORM
The EMBO Journal - Author Guidelines
EMBO Reports - Author Guidelines
Molecular Systems Biology - Author Guidelines
EMBO Molecular Medicine - Author Guidelines

Reporting Checklist for Life Science Articles (updated January
This checklist is adapted from Materials Design Analysis Reporting (MDAR) Checklist for Authors. MDAR establishes a minimum set of requirements in transparent reporting in the life sciences (see Statement of Task: 10.31222/osf.io/9sm4x). Please follow the journal's guidelines in preparing your
Please note that a copy of this checklist will be published alongside your article.

Abridged guidelines for figures
1. Data
The data shown in figures should satisfy the following conditions:
- the data were obtained and processed according to the field's best practice and are presented to reflect the results of the experiments in an accurate and unbiased manner.
- ideally, figure panels should include only measurements that are directly comparable to each other and obtained with the same assay.
- plots include clearly labeled error bars for independent experiments and sample sizes. Unless justified, error bars should not be shown for technical
- if n<5, the individual data points from each experiment should be plotted. Any statistical test employed should be justified.
- Source Data should be included to report the data underlying figures according to the guidelines set out in the authorship guidelines on Data

2. Captions
Each figure caption should contain the following information, for each panel where they are relevant:
- a specification of the experimental system investigated (eg cell line, species name).
- the assay(s) and method(s) used to carry out the reported observations and measurements.
- an explicit mention of the biological and chemical entity(ies) that are being measured.
- an explicit mention of the biological and chemical entity(ies) that are altered/varied/perturbed in a controlled manner.
- the exact sample size (n) for each experimental group/condition, given as a number, not a range;
- a description of the sample collection allowing the reader to understand whether the samples represent technical or biological replicates (including how many animals, litters, cultures, etc.).
- a statement of how many times the experiment shown was independently replicated in the laboratory.
- definitions of statistical methods and measures:
  - common tests, such as t-test (please specify whether paired vs. unpaired), simple χ^2 tests, Wilcoxon and Mann-Whitney tests, can be unambiguously identified by name only, but more complex techniques should be described in the methods section;
  - are tests one-sided or two-sided?
  - are there adjustments for multiple comparisons?
  - exact statistical test results, e.g., P values = x but not P values < x;
  - definition of 'center values' as median or average;
  - definition of error bars as s.d. or s.e.m.

Please complete ALL of the questions below.
Select "Not Applicable" only when the requested information is not relevant for your study.

Materials

Newly Created Materials	Information included in the manuscript?	In which section is the information available? (Reagents and Tools Table, Materials and Methods, Figures, Data Availability Section)
New materials and reagents need to be available; do any restrictions apply?	Yes	Materials and methods

Antibodies	Information included in the manuscript?	In which section is the information available? (Reagents and Tools Table, Materials and Methods, Figures, Data Availability Section)
For **antibodies** provide the following information:<br>- Commercial antibodies: RRID (if possible) or supplier name, catalogue number and or/clone number<br>- Non-commercial: RRID or citation	Yes	Materials and methods

DNA and RNA sequences	Information included in the manuscript?	In which section is the information available? (Reagents and Tools Table, Materials and Methods, Figures, Data Availability Section)
**Short novel DNA or RNA including primers, probes**: provide the sequences.	Not Applicable	

Cell materials	Information included in the manuscript?	In which section is the information available? (Reagents and Tools Table, Materials and Methods, Figures, Data Availability Section)
**Cell lines:** Provide species information, strain. Provide accession number in repository **OR** supplier name, catalog number, clone number, and/**OR** RRID.	Yes	Materials and methods
**Primary cultures:** Provide species, strain, sex of origin, genetic modification status.	Yes	Materials and methods
Report if the cell lines were recently **authenticated** (e.g., by STR profiling) and tested for mycoplasma contamination.	Yes	Materials and methods

Experimental animals	Information included in the manuscript?	In which section is the information available? (Reagents and Tools Table, Materials and Methods, Figures, Data Availability Section)
**Laboratory animals or Model organisms:** Provide species, strain, sex, age, genetic modification status. Provide accession number in repository **OR** supplier name, catalog number, clone number, **OR** RRID.	Yes	Materials and methods
**Animal observed in or captured from the field:** Provide species, sex, and age where possible**.**	Not Applicable	
Please detail **housing and husbandry conditions**.	Yes	Materials and methods

Plants and microbes	Information included in the manuscript?	In which section is the information available? (Reagents and Tools Table, Materials and Methods, Figures, Data Availability Section)
**Plants:** provide species and strain, ecotype and cultivar where relevant, unique accession number if available, and source (including location for collected wild specimens).	Not Applicable	
**Microbes:** provide species and strain, unique accession number if available, and source.	Not Applicable	

Human research participants	Information included in the manuscript?	In which section is the information available? (Reagents and Tools Table, Materials and Methods, Figures, Data Availability Section)
If collected and within the bounds of privacy constraints report on age, sex and gender or ethnicity for all study participants.	Not Applicable	

Core facilities	Information included in the manuscript?	In which section is the information available? (Reagents and Tools Table, Materials and Methods, Figures, Data Availability Section)
If your work benefited from core facilities, was their service mentioned in the acknowledgments section?	Yes	Acknowledgments

Design

Study protocol	Information included in the manuscript?	In which section is the information available? (Reagents and Tools Table, Materials and Methods, Figures, Data Availability Section)
If study protocol has been **pre-registered, provide DOI in the manuscript**. For clinical trials, provide the trial registration number **OR** cite DOI.	Not Applicable	
Report the **clinical trial registration number** (at ClinicalTrials.gov or equivalent), where applicable.	Not Applicable	

Laboratory protocol	Information included in the manuscript?	In which section is the information available? (Reagents and Tools Table, Materials and Methods, Figures, Data Availability Section)
Provide DOI OR other citation details if **external detailed step-by-step protocols** are available.	Yes	Materials and methods

Experimental study design and statistics	Information included in the manuscript?	In which section is the information available? (Reagents and Tools Table, Materials and Methods, Figures, Data Availability Section)
Include a statement about **sample size** estimate even if no statistical methods were used.	Not Applicable	
Were any steps taken to minimize the effects of subjective bias when allocating animals/samples to treatment (e.g. **randomization procedure**)? If yes, have they been described?	Yes	Materials and methods
Include a statement about **blinding** even if no blinding was done.	Yes	Materials and methods
Describe **inclusion/exclusion criteria** if samples or animals were excluded from the analysis. Were the criteria pre-established?<br><br>If sample or data points were omitted from analysis, report if this was due to attrition or intentional exclusion and provide justification.	Yes	Materials and methods
For every figure, are **statistical tests** justified as appropriate? Do the data meet the assumptions of the tests (e.g., normal distribution)? Describe any methods used to assess it. Is there an estimate of variation within each group of data? Is the variance similar between the groups that are being statistically compared?	Yes	Results / Captions / Materials and methods

Sample definition and in-laboratory replication	Information included in the manuscript?	In which section is the information available? (Reagents and Tools Table, Materials and Methods, Figures, Data Availability Section)
In the figure legends: state number of times the experiment was **replicated** in laboratory.	Yes	Figure legends
In the figure legends: define whether data describe **technical or biological replicates**.	Yes	Figure legends

Ethics

Ethics	Information included in the manuscript?	In which section is the information available? (Reagents and Tools Table, Materials and Methods, Figures, Data Availability Section)
Studies involving **human participants**: State details of **authority granting ethics approval** (IRB or equivalent committee(s), provide reference number for approval.	Not Applicable	
Studies involving **human participants**: Include a statement confirming that **informed consent** was obtained from all subjects and that the experiments conformed to the principles set out in the WMA Declaration of Helsinki and the Department of Health and Human Services Belmont Report.	Not Applicable	
Studies involving **human participants:** For publication of **patient photos**, include a statement confirming that consent to publish was obtained.	Not Applicable	
Studies involving experimental **animals**: State details of **authority granting ethics approval** (IRB or equivalent committee(s), provide reference number for approval. Include a statement of compliance with ethical regulations.	Yes	Materials and methods
Studies involving **specimen and field samples:** State if relevant **permits** obtained, provide details of authority approving study; if none were required, explain why.	Not Applicable	

Dual Use Research of Concern (DURC)	Information included in the manuscript?	In which section is the information available? (Reagents and Tools Table, Materials and Methods, Figures, Data Availability Section)
Could your study fall under dual use research restrictions? Please check biosecurity documents and list of **select agents and toxins** (CDC): https://www.selectagents.gov/sat/list.htm	Not Applicable	
If you used a select agent, is the security level of the lab appropriate and reported in the manuscript?	Not Applicable	
If a study is subject to dual use research of concern regulations, is the name of the **authority granting approval and reference number** for the regulatory approval provided in the manuscript?	Not Applicable	

Reporting

The MDAR framework recommends adoption of discipline-specific guidelines, established and endorsed through community initiatives. Journals have their own policy about requiring specific guidelines and recommendations to complement MDAR.

Adherence to community standards	Information included in the manuscript?	In which section is the information available? (Reagents and Tools Table, Materials and Methods, Figures, Data Availability Section)
State if relevant guidelines or checklists (e.g., **ICMJE, MIBBI, ARRIVE, PRISMA**) have been followed or provided.	Not Applicable	
For **tumor marker prognostic studies,** we recommend that you follow the **REMARK** reporting guidelines (see link list at top right). See author guidelines, under 'Reporting Guidelines'. Please confirm you have followed these guidelines.	Not Applicable	
For **phase II and III randomized controlled trials**, please refer to the **CONSORT** flow diagram (see link list at top right) and submit the CONSORT checklist (see link list at top right) with your submission. See author guidelines, under 'Reporting Guidelines'. Please confirm you have submitted this list.	Not Applicable	

Data Availability

Data availability	Information included in the manuscript?	In which section is the information available? (Reagents and Tools Table, Materials and Methods, Figures, Data Availability Section)
Have **primary datasets** been deposited according to the journal's guidelines (see 'Data Deposition' section) and the respective accession numbers provided in the Data Availability Section?	Yes	Data Availability
Were **human clinical and genomic datasets** deposited in a public access-controlled repository in accordance to ethical obligations to the patients and to the applicable consent agreement?	Not Applicable	
Are **computational models** that are central and integral to a study available without restrictions in a machine-readable form? Were the relevant accession numbers or links provided?	Not Applicable	
If publicly available data were reused, provide the respective **data citations in the reference list**.	Yes	Data Availability
